# County-level spatiotemporal patterns, forecasting, and non-fatal burden of human brucellosis in Hulunbuir, China, 2016–2025

**DOI:** 10.3389/fpubh.2026.1876534

**Published:** 2026-07-09

**Authors:** Jianglong Jiang, Dan Xu, Xianlin Qiao, Xiaomeng Li, Zehao Li, Ping Qu, Songwang Zhang

**Affiliations:** 1 Hulunbuir Second People’s Hospital (Hulunbuir Infectious Disease Hospital), Hulunbuir, Inner Mongolia, China; 2 Zhalantun Vocational College, Hulunbuir, Inner Mongolia, China; 3 College of Information and Electrical Engineering, China Agricultural University, Beijing, China

**Keywords:** approximate non-fatal burden, Bayesian smoothing, county-level heterogeneity, forecasting, Hulunbuir, human brucellosis, population-adjusted incidence

## Abstract

**Background:**

Human brucellosis remains an important zoonotic public health problem in northern China, especially in pastoral and agro-pastoral areas. County-level evidence is needed to distinguish large absolute service burden from high population-adjusted risk and to support seasonal preparedness.

**Methods:**

We conducted a retrospective surveillance-based county-level analysis integrating descriptive epidemiology, population-adjusted spatial analysis, Bayesian smoothing, short-term forecasting, and approximate non-fatal burden estimation. County-level population denominators were available for 2016–2024. Because 2025 records had a high deletion rate and lacked reliable county-level denominator linkage, analyses involving 2025 were treated as provisional reported-case analyses.

**Results:**

After excluding 2,615 deleted records, 12,220 reported cases were included; the denominator-linked 2016–2024 panel contained 10,824 cases and 21,892,827 person-years. Annual crude incidence peaked in 2021 at 68.96 per 100,000 population. The largest absolute burdens occurred in Zhalantun City, Arun Banner, and Morin Dawa Daur Autonomous Banner, whereas the highest population-adjusted incidence rates were observed in New Barag Right Banner, New Barag Left Banner, and Chen Barag Banner. Monte Carlo scan-statistic testing supported the leading population-adjusted excess windows in Arun Banner in 2019, Zhalantun City in 2021, and New Barag Right Banner in 2022 (all Monte Carlo *p* < 0.001). BYM smoothing produced consistent high-relative-risk areas, and posterior medians, credible intervals, and exceedance probabilities are reported in the Supplementary materials. In rolling-origin validation, SARIMA achieved the lowest average monthly MAE and MAPE; LightGBM produced a marginally lower mean annual total error across all four folds, but this advantage was not retained after excluding the provisional 2025 fold. Forecasting was interpreted as short-term preparedness support, not as evidence of a single superior model. Approximate non-fatal burden estimates varied across disability-weight and duration assumptions; the upper-bound proxy scenario produced approximately 1,530 DALYs for 2016–2025 and approximately 1,350 DALYs when provisional 2025 records were excluded.

**Conclusion:**

Human brucellosis in Hulunbuir showed persistent seasonality and marked county-level heterogeneity. Formal population-adjusted interpretation was strongest for 2016–2024, while 2025 results should be read as provisional reported-case evidence.

## 1 Introduction

Brucellosis is a bacterial zoonosis caused by *Brucella* spp. and is transmitted mainly through direct contact with infected animals or animal tissues, or through the consumption of contaminated animal products (1–3). Beyond acute infection, human brucellosis may involve prolonged symptoms, recurrent illness, delayed diagnosis, and chronic functional impairment, particularly among occupationally exposed populations (1, 2, 4). Its control therefore requires both timely human surveillance and coordinated prevention at the livestock-human interface.

In China, human brucellosis remains concentrated in northern pastoral and agro-pastoral regions, and Inner Mongolia continues to report substantial transmission (2, 3). Hulunbuir is a particularly relevant setting for county-level public health investigation because it combines extensive grassland, large cattle and sheep populations, cross-regional livestock-related activities, and a large farming and herding population. These characteristics may generate marked local heterogeneity that can be masked by city-level or regional averages.

Previous studies have often examined descriptive epidemiology or a single analytic dimension of brucellosis. Fewer studies have brought together long-term county-level surveillance, population-adjusted spatial heterogeneity, exploratory local spatial signals, Bayesian spatial smoothing, short-term forecasting, and approximate non-fatal burden estimation in the same setting. For a geographically large and epidemiologically heterogeneous area such as Hulunbuir, this combined approach can add practical detail by distinguishing service workload, relative burden, stabilized small-area estimates, preparedness timing, and burden communication.

This integrated county-level framework was designed to distinguish service workload, population-adjusted relative burden, stabilized small-area estimates, preparedness timing, and approximate non-fatal disease burden. The decision-support role of each analytic component is summarized in Supplementary Table S8.

Using NNDRS surveillance data from 2016 to 2025 and county-level population denominators for 2016–2024, this study aimed to characterize temporal, spatial, and population patterns of human brucellosis in Hulunbuir; distinguish absolute reported burden from population-adjusted incidence and O/E excess; identify population-adjusted excess windows using LLR rankings and Monte Carlo scan-statistic *p*-values, and assess exploratory local spatial signals; evaluate short-term forecasting performance for preparedness; and estimate approximate non-fatal burden. The findings are intended to support county-specific surveillance review, seasonal preparedness, and targeted prevention planning in pastoral and agro-pastoral areas.

## 2 Materials and methods

### 2.1 Study design and reporting guideline

This retrospective surveillance-based observational study used routinely collected human brucellosis surveillance data and was reported with reference to the STROBE guideline and, where applicable, the RECORD guideline for studies using routinely collected health data.

### 2.2 Study area

Hulunbuir is a prefecture-level city in northeastern Inner Mongolia, China, bordering Russia and Mongolia. It comprises 13 county-level administrative units: Hailar District, Arun Banner, Morin Dawa Daur Autonomous Banner, Oroqen Autonomous Banner, Ewenki Autonomous Banner, Chen Barag Banner, New Barag Left Banner, New Barag Right Banner, Manzhouli City, Yakeshi City, Zhalantun City, Erguna City, and Genhe City. The region includes extensive grassland and pastoral or agro-pastoral production systems, with frequent human activities related to animal husbandry and animal-product handling. These features make Hulunbuir an appropriate setting for examining county-level heterogeneity in human brucellosis surveillance and prevention.

### 2.3 Data source and case inclusion

We obtained all reported human brucellosis records for Hulunbuir from the National Notifiable Disease Reporting System (NNDRS) for January 1, 2016 to December 31, 2025. The annual source files included sex, age, date of birth, current residential address code, occupation, case classification, onset date, diagnosis date, reporting unit, and audit status. Case inclusion followed the national diagnostic criteria for human brucellosis (WS 269–2019) and the final surveillance audit status (5). For 2016–2018 records, the final NNDRS-audited case classification was retained because retrospective reclassification using WS 269–2019 was not possible from the available de-identified surveillance fields. Records marked as deleted were excluded, and records with valid final or corrected status were retained. The analysis used de-identified secondary surveillance data and did not include direct contact with participants.

### 2.4 Population denominators and analytic scope

County-level annual household-registered population denominators were assembled for 2015–2024 from statistical yearbook and public security household-registration tables. Candidate 2025 population records were available from the source population table, but their population-standard annotations were inconsistent; therefore, they were not used in formal population-corrected analyses. Matched county-year denominators were complete for the 13 analytic counties during 2016–2024. The denominator-linked analytic panel included 117 county-year observations, 21,892,827 person-years, and 10,824 reported cases.

For each county-year unit, crude incidence was calculated as reported cases divided by county-year population and expressed per 100,000 population. Expected counts for denominator-based scan and BYM analyses were calculated as population-proportional expected counts within each calendar year: $E_{\mathrm{it}}=P_{\mathrm{it}}\times C_{t}/P_{t}$ . where $E_{\mathrm{it}}$ is the expected count for county i in year t , $P_{\mathrm{it}}$ is the county-year population, $C_{t}$ is the total cases in year t , and $P_{t}$ is the total population in year t . Because matched 2025 county-level denominators were not available, 2025 records were retained for descriptive temporal summaries, provisional forecasting evaluation, and approximate non-fatal burden estimation, but excluded from denominator-based county risk analyses. To assess potential household-registration denominator bias, we inflated the exposed-population denominator for selected pastoral/high-mobility counties by 10, 20, and 30% and recalculated cumulative county-level incidence rankings for 2016–2024 (Supplementary Table S5). For this scenario analysis, five pastoral or high-mobility counties with plausible denominator mismatch were selected *a priori*: New Barag Right Banner, New Barag Left Banner, Chen Barag Banner, Ewenki Autonomous Banner, and Erguna City.

### 2.5 Data cleaning and quality control

Excel serial date fields were converted to calendar dates. Residential address codes were mapped to the 13 county-level units of Hulunbuir using the first six digits of the national administrative code. Records with onset dates outside the study period were excluded from temporal aggregation. Internal consistency across annual files was checked so that county assignment and time aggregation followed harmonized coding rules.

A total of 14,835 original records were processed. Annual inclusion and deletion flows are provided in Supplementary Table S1. After 2,615 deleted records were excluded, 12,220 cases remained in the analytic dataset. The deletion rate for 2025 records (839 of 2,235; 37.5%) was substantially higher than rates for 2016–2024 (range 8.4–26.3%; mean 13.7%). Retained and deleted 2025 records were compared using available raw-file fields, including county, onset month, sex, age group, occupation, case classification, and reporting unit type (Supplementary Table S2). Because deleted cards had not passed final audit and included duplicate or non-analytic records, the comparison cannot establish full analytic comparability. Accordingly, all 2025-related analyses were interpreted as provisional reported-case evidence rather than formal denominator-based incidence or burden evidence.

### 2.6 Descriptive epidemiological analysis

Annual and monthly case counts were summarized to describe long-term trends and seasonality. County-level cumulative counts were used to characterize absolute reported burden. For 2016–2024, county-year population denominators were used to calculate annual crude incidence, cumulative county-level person-year incidence, and differences between absolute case-burden rankings and population-adjusted incidence rankings. Sex and occupation were summarized to describe the demographic and occupational distribution of reported cases.

### 2.7 Spatiotemporal scan analysis

We used a custom Poisson scan-statistic framework to rank county-year units and consecutive 2-year windows with observed counts above population-based expected counts (6, 7). The primary analysis was restricted to 2016–2024 and used county-year population denominators. For each window, we calculated observed counts, expected counts, relative risk (RR), and the Poisson log-likelihood ratio (LLR). Sensitivity checks using maximum population windows of 50, 30, and 25% produced the same leading windows because all single-county windows represented less than 25% of the study population. To obtain scan-statistic *p*-values, we generated 9,999 Monte Carlo datasets under the null model in which each calendar year’s total case count was fixed and county counts were randomly allocated according to that year’s county population shares. For each simulated dataset, all single-year county windows and consecutive 2-year county windows were recalculated, and the maximum LLR across all evaluated windows was retained. The Monte Carlo *p*-value for each observed window was calculated as (1 + the number of simulated maximum LLRs greater than or equal to the observed LLR)/(9,999 + 1), providing a global scan-statistic *p*-value across the evaluated window set.

This population-based expected-count specification replaced an earlier proportional expected-count approach based on cumulative county case contribution. The revised method better matches the epidemiological interpretation of relative excess incidence because it evaluates whether observed cases exceed what would be expected from county-year population size. The previous proportional method was not retained as a main result and was moved to the Supplementary materials as a method-comparison note (Supplementary Table S10).

Annual local Getis-Ord Gi* statistics were calculated for each county to characterize local spatial clustering (8). County adjacency was defined using a rook contiguity matrix. Because annual Gi* statistics involved 117 county-year comparisons (13 counties × 9 years), we applied the Benjamini–Hochberg false discovery rate (FDR) correction to one-sided upper-tail Gi* *p*-values. After FDR correction, no county-year unit reached *q* < 0.05, consistent with limited statistical power given the small number of counties (*n* = 13). The yearly Gi* *Z*-score series for each county was assessed using the Mann–Kendall trend test (9, 10). The Gi*–Mann–Kendall component was retained as exploratory hotspot-evolution evidence, and counties with no FDR-significant annual Gi* signal were not interpreted as confirmatory persistent hotspots. Full annual rook-adjacency Gi* outputs and sensitivity analyses using inverse-distance-weighted and K-nearest-neighbor (*K* = 3) weight matrices are reported in the Supplementary Tables S11, S12. Under rook adjacency, only a few uncorrected upper-tail signals were observed, mainly in New Barag Left Banner in 2016, 2017, 2023, and 2024, and in New Barag Right Banner and Manzhouli City in 2017. Sensitivity analyses using KNN = 3 and inverse-distance weights produced some additional uncorrected signals, but none remained FDR-significant. Therefore, Gi* results were retained only as exploratory local spatial-signal diagnostics.

### 2.8 Bayesian BYM spatiotemporal modeling

To reduce instability from small-area fluctuation and account for spatial dependence, we fitted a Bayesian Besag–York–Mollié (BYM)-type spatiotemporal model to county-year counts for 2016–2024 (11). For county i and year t, the observed count $O_{\mathrm{it}}$ was assumed to follow a Poisson distribution, $\;O_{\mathrm{it}}\sim \text{Poisson}(E_{\mathrm{it}}\times \theta_{\mathrm{it}})$ , where E_it is the population-derived expected count and θ_it is the relative risk. The log-risk was modeled as $\log(\theta_{\mathrm{it}})=\alpha +u_{i}+v_{i}+\gamma_{t}+\delta_{t}$ , where u_i denotes the structured proper-CAR spatial effect, v_i denotes the exchangeable county effect, γ_t denotes the first-order random-walk temporal effect, and δ_t denotes the exchangeable year effect. Posterior exceedance probabilities were calculated as *P*( $\theta_{\mathrm{it}}$ > 1) and P( $\theta_{\mathrm{it}}$ > 1.5). The intercept was assigned a Normal(0, 2) prior, and the standard deviations of the spatial, county, and temporal random effects were each assigned a HalfNormal(0.7) prior: $\alpha \sim \text{Normal}(0,2),\sigma_{u},\sigma_{v},\sigma_{\gamma },\sigma_{\delta }\sim \text{HalfNormal}(0.7).$

The structured and unstructured spatial effects were modeled as separate variance components rather than through a BYM2 mixing-weight parameter, so no BYM2 phi mixing parameter was estimated.

The final BYM rerun used real rook adjacency from the county GeoJSON and a non-centered proper-CAR spatial parameterization with rho fixed at 0.99, retaining the population-based expected-count offset. The fixed rho value was used to favor spatially structured smoothing in this small-area setting with only 13 counties, where the BYM model was used primarily for stabilization rather than for decomposing structured and unstructured spatial variance. The final run used 4 chains with 2,000 warm-up and 2,000 post-warm-up draws per chain (target_accept = 0.99; maximum tree depth = 15; random seed = 20,260,507), and the complete posterior chain was saved for diagnostic review. The rerun showed no divergent transitions and no maximum-tree-depth hits; the maximum Rhat across monitored parameters was 1.000, with minimum bulk ESS of 2,208 and minimum tail ESS of 1,580. Posterior mean, posterior median, 95% credible interval, *P*(RR > 1), and *P*(RR > 1.5) were summarized for each county (Supplementary Table S6). Sensitivity analyses using alternative rho values (0.5, 0.8, 0.95, and 0.99) under rook adjacency and an additional queen-adjacency check at rho = 0.99 showed that the leading high-risk counties remained stable; New Barag Right Banner remained first in all settings (Supplementary Table S7).

### 2.9 Short-term forecasting and rolling-origin validation

Monthly case counts from January 2016 to December 2025 were aggregated by onset date and analyzed as a univariate forecasting series. Because the forecasting series was based on onset-month aggregation, its annual totals may differ slightly from county-year totals summarized by administrative annual files for spatial analyses. Forecast accuracy was evaluated using mean absolute error (MAE), root mean square error (RMSE), mean absolute percentage error (MAPE), and absolute percentage error in annual total counts. Mean annual total error was calculated as the mean across validation folds of the absolute difference between predicted and observed annual totals divided by the observed annual total.

A rolling-origin validation was used to evaluate out-of-sample forecasting performance. The training window started in January 2016 and expanded by 1 year at each origin: models trained on 2016–2021 forecasted 2022, models trained on 2016–2022 forecasted 2023, models trained on 2016–2023 forecasted 2024, and models trained on 2016–2024 forecasted 2025. The 2025 fold was treated as a provisional benchmark because of the high deletion rate in that file. Horizon-level forecast errors, available prediction-interval diagnostics, and sensitivity results excluding the provisional 2025 fold are provided in Supplementary Tables S13, S14, S16. For each origin, SARIMA, Prophet, and LightGBM generated 12-month-ahead forecasts (12–15). For LightGBM recursive forecasting, the prediction history was initialized using only observed monthly counts available before the forecast origin. For each horizon h = 1,…,12, lagged features were generated from the current history; if a lag referred to a month already predicted within the same forecast horizon, the previously predicted value was used, otherwise the observed historical value was used. After each step, the predicted count was appended to the history and used for subsequent steps. Thus, no observed values from the target year were used to construct lagged features for later forecast months. Additional comparisons with Holt-Winters, Poisson GLM, FITS, TimesFM, and Chronos-2 are provided in Supplementary Table S9 because these models were exploratory and did not alter the decision-support interpretation (16–18).

### 2.10 DALY estimation

Because no valid death records were identified in the cleaned surveillance table and no independent mortality linkage was available, years of life lost (YLL) were set to zero for this surveillance-based analysis. This should not be interpreted as evidence that fatal brucellosis did not occur. DALYs were therefore equivalent to YLDs and were interpreted as approximate non-fatal burden estimates. Disease burden was estimated using YLD = incident cases × disability weight × duration, following the general Global Burden of Disease framework for non-fatal burden estimation (19, 20). The brucellosis-specific disability-weight scenarios were treated as primary disease-specific scenarios, whereas the GBD infectious-disease post-acute proxy was interpreted as an upper-bound proxy scenario.

Because locally observed disability duration and long-term outcomes were unavailable, scenario-based sensitivity analyses varied both disability weight and disease duration. Disability weights included brucellosis-specific values (DW = 0.150 and 0.190) and GBD proxy values (DW = 0.219 and 0.250), informed by published disability-weight sources and methodological discussions of brucellosis burden estimation (21–23), and disease durations included 3 months, 6 months, and 12 months. Analyses were also summarized including and excluding provisional 2025 records (Supplementary Tables S3, S4).

### 2.11 Software and mapping

Data cleaning, aggregation, and statistical analyses were conducted in Python. Spatial linkage and mapping used county boundary data from GADM. Data processing used pandas and numpy; spatial data handling used geopandas; spatial autocorrelation and local statistics used libpysal and esda; time-series models used statsmodels, prophet, lightgbm, and related forecasting libraries. Bayesian modeling was implemented using PyMC, and convergence diagnostics were summarized using ArviZ. Package versions, sampler settings, and key model settings are summarized in the reproducibility supplement (Supplementary Table S15).

## 3 Results

### 3.1 Temporal trend and annual incidence

**Figure 1:**
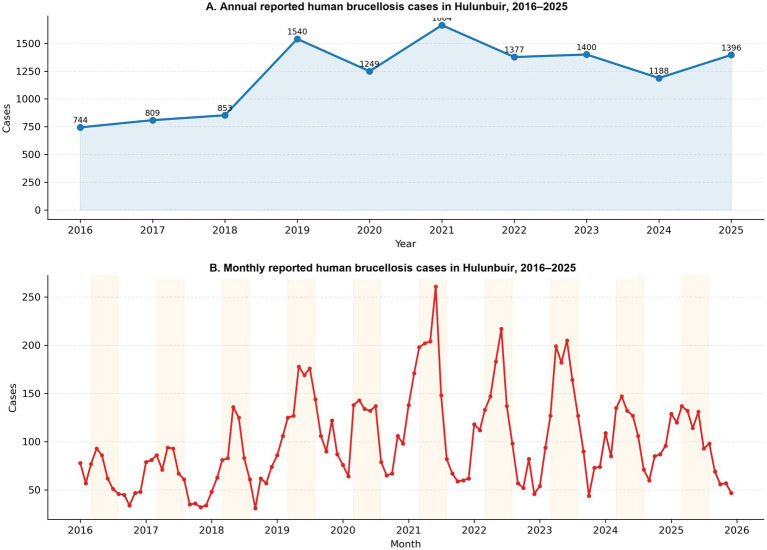
Temporal trend and seasonality of reported human brucellosis cases in Hulunbuir, 2016–2025. (A) Annual reported cases from 2016 to 2025. (B) Monthly distribution of reported cases from 2016 to 2025. The 2025 counts are shown as provisional reported-case evidence because the 2025 file had a high deletion rate and lacked reliable county-level denominator linkage.

After data cleaning, 12,220 human brucellosis cases reported in Hulunbuir during 2016–2025 were included in the descriptive analysis. Annual case counts increased from 744 in 2016 to 1,540 in 2019, declined to 1,249 in 2020, rose again to 1,664 in 2021, and then remained relatively high during 2022–2025, although 2025 should be interpreted as provisional reported-case evidence because of the high deletion rate (Figure 1).

For the denominator-linked period of 2016–2024, the analytic panel covered 21,892,827 person-years and 10,824 cases. Annual crude incidence increased from 29.71 per 100,000 population in 2016 to 62.57 per 100,000 in 2019, reached the highest level in 2021 at 68.96 per 100,000, and remained above 50 per 100,000 during 2022–2024 (Table 1). Monthly counts showed a clear seasonal pattern. Cases generally increased from March, remained elevated through spring and early summer, and declined after July, indicating that March to July was the main high-incidence period in the surveillance series (Figure 1).

**Table 1:** Annual data cleaning and crude incidence, Hulunbuir, 2016–2025, with denominator-based incidence restricted to 2016–2024.

Year	Original records	Deleted records	Included cases	Population	Crude incidence per 100,000
2016	815	71	744	2,503,810	29.71
2017	922	113	809	2,492,769	32.45
2018	996	143	853	2,478,157	34.42
2019	1,681	141	1,540	2,461,263	62.57
2020	1,364	115	1,249	2,439,278	51.20
2021	1,842	178	1,664	2,413,007	68.96
2022	1,663	286	1,377	2,392,524	57.55
2023	1,704	304	1,400	2,366,324	59.16
2024	1,613	425	1,188	2,345,695	50.65
2025	2,235	839	1,396	–	–

### 3.2 Spatial distribution: absolute burden versus population-adjusted incidence

County-level heterogeneity differed substantially according to whether the distribution was summarized by absolute case counts or by population-adjusted incidence. During 2016–2024, the largest absolute reported burdens were recorded in Zhalantun City (2,726 cases), Arun Banner (2,301), and Morin Dawa Daur Autonomous Banner (1,389), indicating substantial service needs for surveillance, diagnosis, and case management. After population correction, smaller pastoral county-level units became more prominent: the highest person-year incidence rates were observed in New Barag Right Banner (159.45 per 100,000), New Barag Left Banner (93.83), Chen Barag Banner (87.60), Arun Banner (80.39), and Zhalantun City (75.70) (Table 2).

**Table 2:** County-level absolute burden and population-adjusted incidence, Hulunbuir, 2016–2024.

County	Cases, 2016–2024	Case rank	Mean population	Incidence per 100,000 person-years	Incidence rank
Zhalantun City	2,726	1	400,115	75.70	5
Arun Banner	2,301	2	318,041	80.39	4
Morin Dawa Daur Autonomous Banner	1,389	3	313,079	49.30	8
Hailar District	705	4	286,692	27.32	10
Oroqen Autonomous Banner	645	5	239,829	29.88	9
Ewenki Autonomous Banner	635	6	136,587	51.66	7
Yakeshi City	518	7	314,801	18.28	11
Erguna City	516	8	78,010	73.49	6
New Barag Right Banner	503	9	35,051	159.45	1
Chen Barag Banner	425	10	53,909	87.60	3
New Barag Left Banner	351	11	41,562	93.83	2
Manzhouli City	88	12	87,513	11.17	12
Genhe City	22	13	127,347	1.92	13

**Figure 2:**
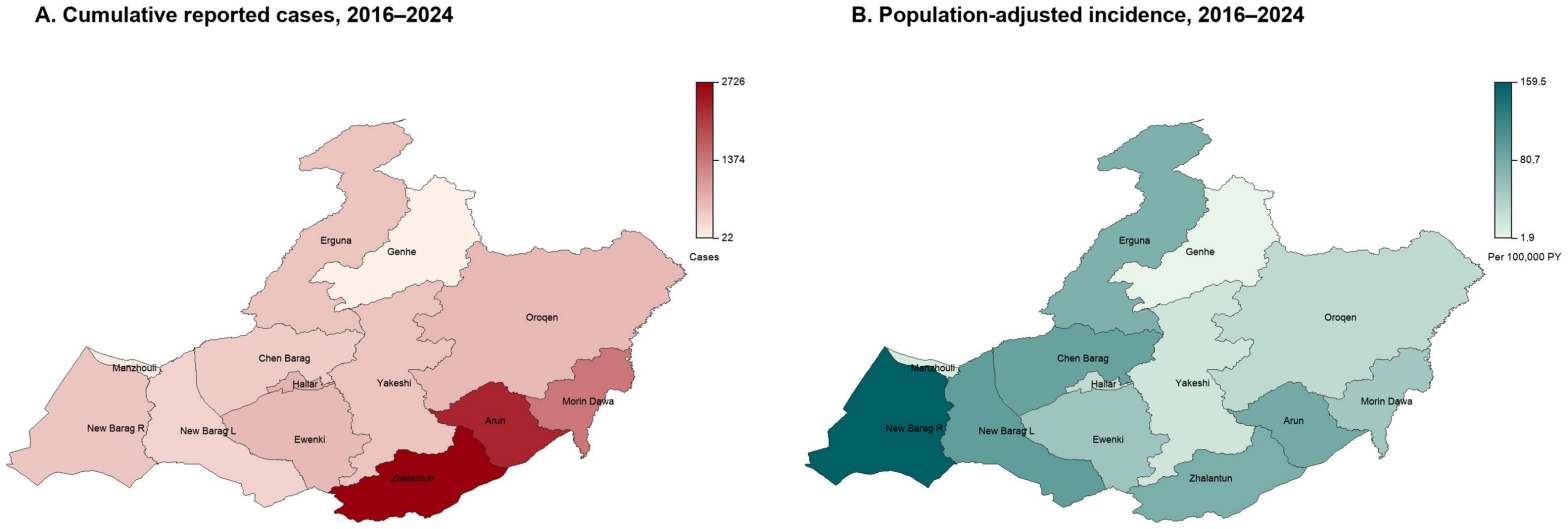
County-level absolute burden and population-adjusted incidence of human brucellosis in Hulunbuir, 2016–2024. **(A)** Cumulative reported cases by county. **(B)** Population-adjusted incidence per 100,000 person-years by county. Population-adjusted incidence maps are restricted to 2016–2024 because matched county-level denominators were not available for 2025.

This re-ranking shows that absolute case counts and population-adjusted incidence answer different public health questions. Absolute counts identify where the largest patient volume and service workload occurred, whereas incidence and O/E excess identify counties whose burden was high relative to population size. This distinction is important because large-population counties should not automatically be interpreted as having the highest relative risk, and smaller counties with sustained excess may require targeted surveillance attention (Figure 2).

In the denominator-inflation sensitivity analysis, New Barag Right Banner remained the highest-incidence county under 10, 20, and 30% exposed-population inflation for selected pastoral or high-mobility counties. However, the ordering of the remaining top-five counties changed across scenarios, indicating that household-registration denominator bias may affect fine ranking among high-incidence counties even though the leading high-incidence signal remained stable (Supplementary Table S5).

### 3.3 Population characteristics

Among the 12,220 included cases, 8,271 were male and 3,949 were female, corresponding to 67.7 and 32.3%, respectively. Farmers accounted for the largest occupational group (7,564 cases; 61.9%), followed by herders (1,884; 15.4%), household or unemployed individuals (1,493, 12.2%), workers (359), students (223), retirees (178), and cadres or staff (131). Overall, reported cases were concentrated in population groups with frequent or plausible livestock-related exposure, although individual exposure histories were not available in the surveillance dataset (Table 3).

**Table 3:** Demographic characteristics of reported cases by sex and occupation, Hulunbuir, 2016–2025.

Characteristic	Category	Cases	Percentage
Sex	Male	8,271	67.7%
Sex	Female	3,949	32.3%
Occupation	Farmers	7,564	61.9%
Occupation	Herders	1,884	15.4%
Occupation	Household/unemployed	1,493	12.2%
Occupation	Workers	359	2.9%
Occupation	Students	223	1.8%
Occupation	Retirees	178	1.5%
Occupation	Cadres/staff	131	1.1%
Occupation	Other/unspecified	388	3.2%

### 3.4 High-incidence county-year units and O/E excess

The highest county-year crude incidence values were concentrated in New Barag Right Banner, reaching 243.00 per 100,000 in 2022, 212.19 per 100,000 in 2023, and 193.79 per 100,000 in 2021. Additional high-incidence signals were observed in Arun Banner in 2019 and Chen Barag Banner in 2023–2024.

The O/E results based on population-proportional expected counts within each calendar year similarly highlighted New Barag Right Banner and New Barag Left Banner. The highest O/E unit was New Barag Right Banner in 2022 (*O* = 85, *E* = 20.13, O/E RR = 4.22), followed by New Barag Right Banner in 2023 (*O* = 74, *E* = 20.63, O/E RR = 3.59) and New Barag Left Banner in 2016 (*O* = 43, *E* = 12.51, O/E RR = 3.44) (Table 4). These values use the same population-based expected-count specification as the scan-statistic windows in Table 5.

**Table 4:** County-year units with the highest observed/expected excess based on population-proportional expected counts within each calendar year, Hulunbuir, 2016–2024.

Rank	County	Year	Cases	Population	Incidence per 100,000	Expected cases	O/E RR
1	New Barag Right Banner	2022	85	34,979	243.00	20.13	4.22
2	New Barag Right Banner	2023	74	34,874	212.19	20.63	3.59
3	New Barag Left Banner	2016	43	42,093	102.15	12.51	3.44
4	New Barag Right Banner	2020	60	35,012	171.37	17.93	3.35
5	New Barag Right Banner	2024	58	34,770	166.81	17.61	3.29
6	New Barag Right Banner	2017	37	35,190	105.14	11.42	3.24
7	New Barag Right Banner	2018	37	35,223	105.04	12.12	3.05
8	New Barag Left Banner	2017	41	42,156	97.26	13.68	3.00

Expected counts were calculated according to county-year population shares, consistent with the population-based scan-statistic specification.

**Table 5:** Population-based spatiotemporal scan-statistic windows with Monte Carlo *p*-values for human brucellosis in Hulunbuir, 2016–2024.

LLR-ranked window	County	Period	O	E	RR	LLR	Monte Carlo p-value
Single-year 1	Arun Banner	2019	466	200.74	2.32	127.20	<0.001
Single-year 2	Zhalantun City	2021	521	273.76	1.90	88.03	<0.001
Single-year 3	New Barag Right Banner	2022	85	20.13	4.22	57.56	<0.001
Two-year 1	Arun Banner	2018–2019	695	311.11	2.23	174.73	<0.001
Two-year 2	Arun Banner	2019–2020	764	364.58	2.10	165.81	<0.001
Two-year 3	Zhalantun City	2020–2021	846	479.22	1.77	114.05	<0.001

### 3.5 Population-based scan-statistic excess windows

**Figure 3:**
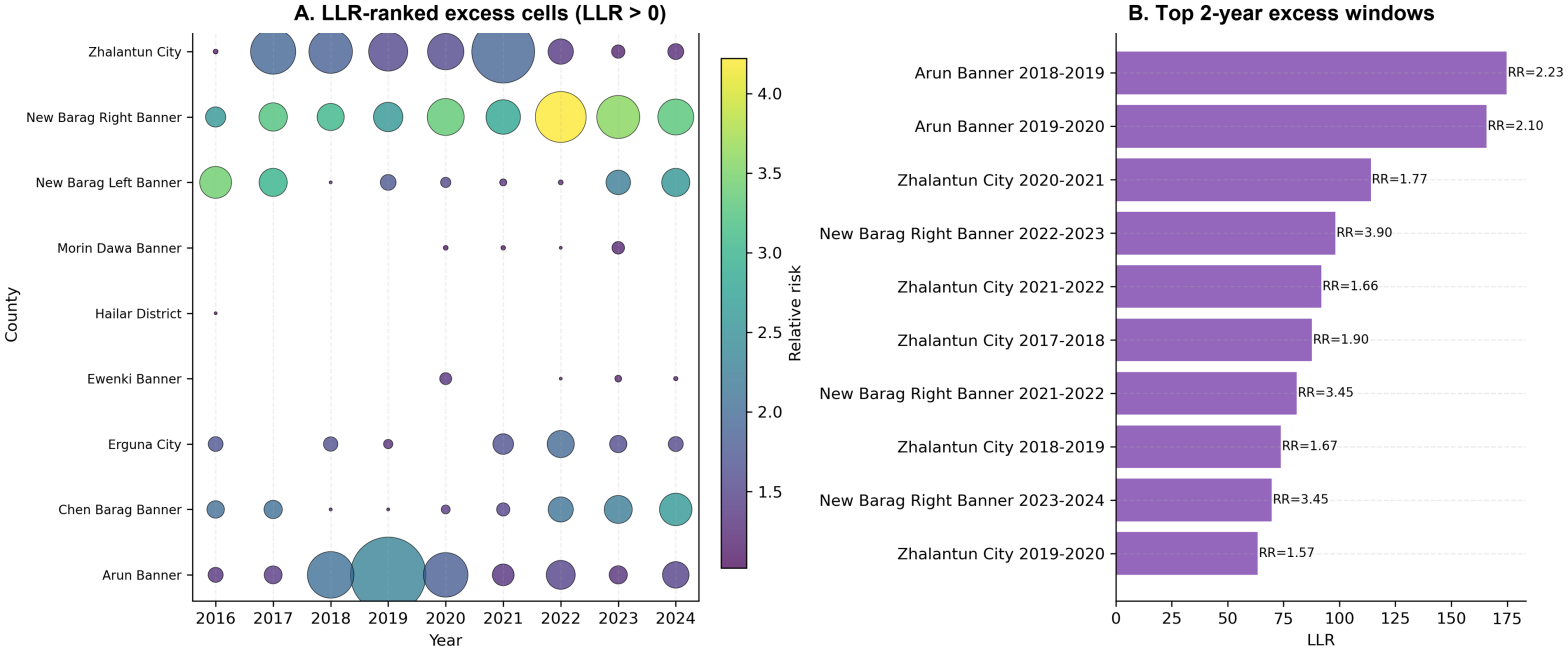
Population-based spatiotemporal scan-statistic windows for human brucellosis in Hulunbuir, 2016–2024. **(A)** LLR-ranked county-year excess units, with bubble size representing LLR and color representing RR. **(B)** Top 2-year excess windows ranked by LLR. Monte Carlo p-values were calculated under a population-proportional null model with annual total cases fixed; leading single-year and 2-year windows in Table 5 had global scan *p*-values < 0.001.

The population-based scan-statistic analysis highlighted several county-year units with elevated observed counts relative to population-based expected counts for 2016–2024. The largest single-year windows by LLR were Arun Banner in 2019 (*O* = 466, *E* = 200.74, RR = 2.32, LLR = 127.20); Zhalantun City in 2021 (*O* = 521, *E* = 273.76, RR = 1.90, LLR = 88.03); New Barag Right Banner in 2022 (*O* = 85, *E* = 20.13, RR = 4.22, LLR = 57.56); Arun Banner in 2018 (*O* = 229, *E* = 110.61, RR = 2.07, LLR = 48.26); and Zhalantun City in 2017 (*O* = 257, *E* = 132.75, RR = 1.94, LLR = 45.52) (Figure 3 and Table 5). In the Monte Carlo scan test, the top three single-year windows reported in Table 5 all had global scan *p*-values < 0.001.

The 2-year window analysis suggested repeated high-LLR population-adjusted excess signals, not isolated annual peaks. The leading windows were Arun Banner in 2018–2019 (*O* = 695, *E* = 311.11, RR = 2.23, LLR = 174.73); Arun Banner in 2019–2020 (*O* = 764, *E* = 364.58, RR = 2.10, LLR = 165.81); Zhalantun City in 2020–2021 (*O* = 846, *E* = 479.22, RR = 1.77, LLR = 114.05); New Barag Right Banner in 2022–2023 (*O* = 159, *E* = 40.76, RR = 3.90, LLR = 98.18); and Zhalantun City in 2021–2022 (*O* = 833, *E* = 500.44, RR = 1.66, LLR = 91.89) (Figure 3 and Table 5). The top three 2-year windows reported in Table 5 also had global scan *p*-values < 0.001.

### 3.6 Exploratory hotspot evolution

The combined Gi* and Mann–Kendall analysis provided an exploratory view of local spatial signals over time. Under rook adjacency, only a few uncorrected upper-tail signals were observed, mainly in New Barag Left Banner in 2016, 2017, 2023, and 2024, and in New Barag Right Banner and Manzhouli City in 2017. Full hotspot outputs and spatial-weight sensitivity analyses are reported in the Supplementary Tables S11, S12. Because no county-year unit remained significant after FDR correction, these findings were retained only as exploratory local spatial-signal diagnostics.

### 3.7 Bayesian spatiotemporal smoothing results

The rerun BYM spatiotemporal model showed satisfactory convergence diagnostics, with no divergent transitions and no maximum-tree-depth hits (max Rhat = 1.000, min bulk ESS = 2,208, min tail ESS = 1,580), using population-based expected counts for 2016–2024. New Barag Right Banner had the highest posterior relative risk (median RR 3.206, 95% CrI 2.935–3.493, P[RR > 1.5] 1.000), followed by New Barag Left Banner, Chen Barag Banner, Arun Banner, and Zhalantun City. County-level posterior means, posterior medians, 95% credible intervals, and exceedance probabilities are provided in Supplementary Table S6.

Given the satisfactory convergence diagnostics, the BYM outputs were interpreted as model-based smoothing estimates, not qualitative sensitivity findings alone. The BYM results are consistent with the main population-adjusted findings: smaller pastoral counties, especially New Barag Right Banner and New Barag Left Banner, had higher relative excess after accounting for population size. Interpretation remains bounded by the available surveillance data and population-denominator scope (2016–2024).

### 3.8 Use of 2025 data

In 2025, 1,396 retained reported cases accounted for 11.42% of the 2016–2025 analytic cases, but the 2025 raw file also contained 839 deleted records and a 37.5% deletion rate. Because of this high deletion rate and the lack of reliable county-level denominator linkage, 2025 was not used to infer formal county-level incidence risk. All 2025-related results should be read as provisional reported-case summaries. The retained-versus-deleted comparison is provided in Supplementary Table S2, but deleted records were not final analytic cases and therefore cannot fully establish comparability.

### 3.9 Forecasting performance, rolling-origin validation, and projected trend

Across the four rolling-origin validation folds, SARIMA achieved the lowest average monthly MAE (23.12 cases/month) and MAPE (23.05%) across 48 forecast months. LightGBM produced a marginally lower mean annual total error than SARIMA in the all-fold summary (13.74% versus 14.01%), but its average monthly MAE and MAPE were higher and recursive multi-step updating can accumulate forecast errors. When the provisional 2025 fold was excluded, SARIMA remained the most stable model, with the lowest average monthly MAE (23.52), MAPE (22.46%), and mean annual total error (12.45%) across the 2022–2024 validation folds (Supplementary Table S16). These results support using forecasting as short-term preparedness evidence rather than a model-ranking exercise (Table 6).

**Table 6:** Forecasting performance in rolling-origin validation; the full provisional 2025 all-model benchmark and the sensitivity analysis excluding the provisional 2025 fold are reported in Supplementary Tables S9, S16.

Evaluation	Model	Folds/months	MAE	RMSE	MAPE	Mean annual total error
Rolling-origin validation	SARIMA	4/48	23.12	28.68	23.05%	14.01%
Rolling-origin validation	Prophet	4/48	31.62	37.64	39.12%	25.19%
Rolling-origin validation	LightGBM	4/48	24.96	32.17	27.53%	13.74%

Additional exploratory 2025 benchmarks are provided in Supplementary Table S9, but these comparisons were not used as primary evidence because the 2025 fold was provisional. The sensitivity analysis excluding the 2025 fold is reported in Supplementary Table S16. Taken together, the forecasting results are best used as short-term preparedness support, not deterministic prediction or a model competition.

For the available SARIMA rolling-origin interval diagnostics, all 48 observed monthly counts fell within the nominal 95% prediction intervals, including the provisional 2025 fold; however, the intervals were wide and this result should not be interpreted as proof of full calibration. Because interval outputs were not available or not comparable for all forecasting models, formal interval-calibration comparison across models was not possible and was treated as a forecasting limitation (Supplementary Table S14).

**Figure 4:**
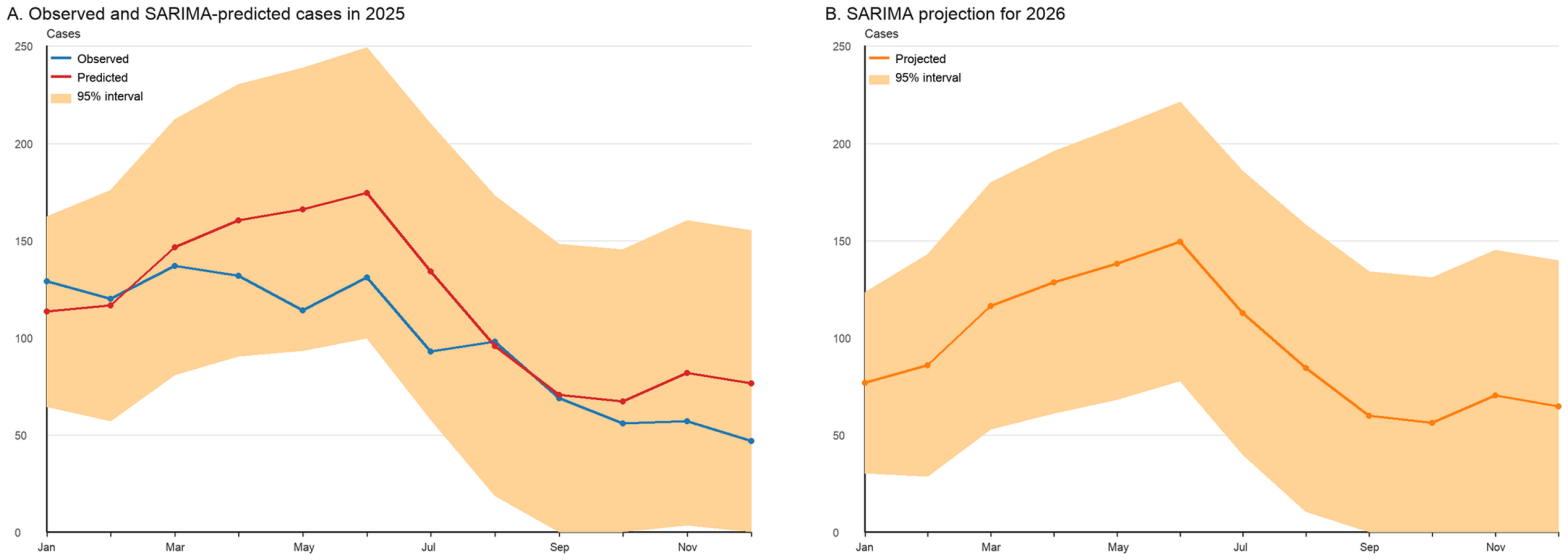
Forecasting performance and 2026 projection for monthly human brucellosis cases in Hulunbuir. **(A)** Observed and SARIMA-predicted monthly cases in 2025. **(B)** SARIMA-projected monthly cases in 2026. The projection panel displays model-based 95% prediction intervals from the fitted SARIMA forecast distribution; negative lower bounds were truncated at zero for graphical display on the case-count scale. The 2026 projection was fitted using the 2016–2025 onset-month series, including provisional 2025 records.

After refitting on the full 2016–2025 series, including provisional 2025 records, the SARIMA model projected a continued seasonal pattern in 2026, with lower counts in January–February, a spring–summer rise in March–June, and lower counts in August–December (Figure 4). These projections are best used as short-term preparedness estimates conditional on historical surveillance patterns.

### 3.10 Disease burden

Under the GBD 2010 post-acute upper-bound proxy scenario (DW = 0.250, duration = 0.5 years), the estimated approximate non-fatal burden was approximately 1,530 DALYs for 2016–2025 and approximately 1,350 DALYs when provisional 2025 records were excluded (Table 7 and Supplementary Table S3). These values represent approximate YLD-based non-fatal burden estimates.

**Table 7:** Scenario-based approximate non-fatal burden estimates for human brucellosis in Hulunbuir.

Scenario	Period	DW × duration	Estimated DALYs
Brucellosis-specific chronic localized	2016–2025	0.150 × 0.50 years	916.5
Brucellosis-specific acute	2016–2025	0.190 × 0.50 years	1,160.9
GBD 2021 post-acute proxy	2016–2025	0.219 × 0.50 years	1,338.1
GBD 2010 post-acute upper-bound proxy	2016–2025	0.250 × 0.50 years	1,527.5
Upper-bound proxy excluding provisional 2025	2016–2024	0.250 × 0.50 years	1,353.0

**Figure 5:**
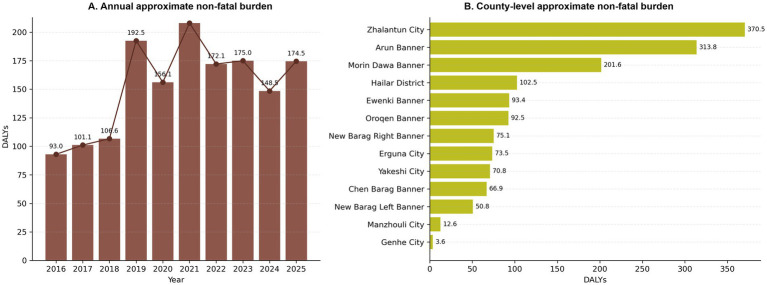
Scenario-based approximate non-fatal burden estimates for human brucellosis in Hulunbuir. **(A)** Annual approximate non-fatal burden measured in DALYs from 2016 to 2025 under the upper-bound proxy scenario. **(B)** County-level approximate non-fatal burden ranking. Estimates are YLD-based because no valid death records were available in the cleaned surveillance table and no independent mortality linkage was available. The GBD 2010 post-acute scenario is interpreted as an upper-bound proxy; estimates involving 2025 include provisional reported-case records.

Across disability-weight and duration assumptions, the estimated burden varied substantially (Supplementary Table S4), confirming that the DALY analysis is an approximate non-fatal burden assessment, not a precise local measurement (Figure 5).

## 4 Discussion

### 4.1 Principal findings

This study examined 10 years of county-level human brucellosis surveillance data from Hulunbuir using descriptive epidemiology, denominator-linked spatial analysis, forecasting, Bayesian smoothing, and burden estimation. Adding county-year population denominators changed the interpretation of spatial heterogeneity by allowing high-workload counties and smaller high-relative-risk counties to be considered together, instead of relying only on the geographic distribution of reported case counts.

This study does not propose a new statistical model, and multi-method analyses of brucellosis have been reported previously. Its contribution is decision-relevant integration: the practical value lies in the points at which the analytic lenses disagree, which no single method can reveal on its own. By absolute case count, the leading counties were Zhalantun City, Arun Banner, and Morin Dawa Daur Autonomous Banner, which define diagnostic and case-management workload. By population-adjusted incidence, the leading counties were the smaller pastoral New Barag Right Banner, New Barag Left Banner, and Chen Barag Banner; New Barag Right Banner ranked first on incidence but far lower on absolute counts, whereas Zhalantun City ranked first on absolute counts but only fifth on incidence. The scan-statistic analysis additionally isolated a time-localized excess in Arun Banner in 2019 (RR = 2.32, global Monte Carlo *p* < 0.001) that neither cumulative ranking captured, and BYM smoothing confirmed that the high incidence in New Barag Right Banner (median RR 3.206, P[RR > 1.5] = 1.000) was not a small-denominator artifact. A single-method analysis would therefore yield a different and potentially misleading allocation: a case-count-only view would overlook the small high-incidence pastoral banners, whereas an incidence-only view would under-resource the high-volume counties that carry the largest patient load. Reconciling these rankings supports a two-track allocation strategy, comprising a service-capacity track for high-volume counties and a risk-investigation track for small high-relative-risk counties, with a seasonal timing layer (acting before the March–July rise) and an anomaly layer (flagging time-localized scan windows such as Arun Banner in 2019 for retrospective review). The decision-support role and the single-method limitation addressed by each analytic component are summarized in Supplementary Table S8.

Human brucellosis remained endemic in Hulunbuir throughout 2016–2025, with a clear seasonal concentration from March to July. In pastoral and agro-pastoral settings, this pattern is epidemiologically plausible because spring and early summer may coincide with lambing and calving seasons, increased handling of livestock and animal products, and more opportunities for occupational exposure. For public health practice, the timing is important: prevention messaging, diagnostic vigilance, occupational protection, and intersectoral preparedness should be strengthened before the annual rise in cases, not after the seasonal peak has developed.

The denominator-linked results also changed the spatial reading of the surveillance data. Zhalantun City, Arun Banner, and Morin Dawa Daur Autonomous Banner contributed the largest numbers of reported cases and therefore represent major workload areas for surveillance, clinical diagnosis, and case management. Smaller pastoral counties became more prominent after population adjustment: New Barag Right Banner, New Barag Left Banner, and Chen Barag Banner had the highest incidence rates and O/E excess.

The scan-statistic analysis added a time-window perspective rather than simply reproducing the cumulative maps. Monte Carlo *p*-values supported the leading single-year and 2-year excess windows, with the strongest signals concentrated in Arun Banner during 2018–2020 and later prominent windows in Zhalantun City and New Barag Right Banner. These spatial signals indicate excess in human surveillance records after accounting for population size, but they do not establish changes in transmission mechanisms.

The 80.5% increase in reported cases from 2018 (853 cases) to 2019 (1,540 cases) was the most abrupt annual change in the study period and warrants specific consideration. The scan-statistic analysis identified Arun Banner in 2019 as the strongest single-year excess signal (*O* = 466, *E* = 200.74, RR = 2.32, LLR = 127.20; global Monte Carlo *p* < 0.001), indicating that this county was an important contributor to the city-wide surge. Several explanations are plausible but cannot be separated with the present human-surveillance data: a true livestock-related outbreak or amplification event, intensified case screening or reporting, changes in diagnostic capacity, or cross-regional livestock movement affecting exposure opportunities. Linkage with animal brucellosis surveillance, livestock movement records, vaccination coverage, and local reporting-policy documentation would be required to distinguish these mechanisms.

The comparison with the earlier proportional expected-count approach is also useful for interpretation. The earlier method effectively asked whether a county-year exceeded its own long-term case contribution; the updated method asks whether observed cases exceed what would be expected from population size. This shift makes the scan-statistic results more consistent with conventional spatial epidemiology and better suited for identifying county-year excess incidence.

The BYM rerun provided a complementary smoothed view of county risk. With the non-centered proper-CAR spatial parameterization, diagnostics were satisfactory and county rankings were consistent with the main spatial results, while posterior medians, credible intervals, and exceedance probabilities retained the uncertainty in the small-area estimates. The rho and adjacency sensitivity analyses also supported the stability of the leading counties, with New Barag Right Banner remaining top-ranked in all tested settings (Supplementary Table S7).

Forecasting contributed a narrower, operational piece of evidence. Rolling-origin validation showed that SARIMA had the lowest average monthly MAE and MAPE across four forecast origins; after the provisional 2025 fold was excluded, SARIMA also had the lowest mean annual total error. LightGBM’s marginal all-fold annual-total advantage should therefore be interpreted cautiously, especially because recursive lag updating can accumulate multi-step forecast errors. For county and city health authorities, the main value of forecasting is not month-by-month certainty, but earlier anticipation of seasonal pressure on surveillance, laboratory testing, clinical diagnosis, and risk communication.

The burden estimates add a different dimension to the surveillance picture. Even when mortality linkage is unavailable, brucellosis can impose a meaningful non-fatal burden through prolonged symptoms, reduced work capacity, and delayed recovery. The upper-bound proxy scenario should not be treated as a single definitive estimate; the two-way sensitivity analysis shows that burden estimates depend strongly on both disability weight and assumed disease duration.

### 4.2 Comparison with previous studies

The overall pattern is consistent with previous evidence that human brucellosis in China is concentrated in northern pastoral and agro-pastoral regions and is closely related to occupational exposure at the livestock-human interface (2, 3, 24). The seasonal concentration observed in Hulunbuir also aligns with reports from endemic pastoral settings in which spring and early summer are high-risk periods for livestock-related contact. The present study extends these observations by showing that, within a single prefecture-level city, county-level rankings can differ substantially depending on whether the analysis focuses on absolute case burden, population-adjusted incidence, or O/E excess.

### 4.3 One health interpretation and evidence gap

The observed spring-to-early-summer concentration of human brucellosis and the concentration of high relative incidence in pastoral counties are compatible with increased livestock-related exposure during lambing, calving, animal handling, livestock movement, and contact with animal products. However, the present study did not include animal infection surveillance, livestock density, livestock vaccination coverage, climate variables, NDVI, land use, or socioeconomic covariates. Therefore, the findings support One Health-oriented monitoring but do not prove a complete animal-environment-human causal pathway.

This distinction is important for interpretation. The analysis identifies where and when human reported cases and population-adjusted excess were concentrated, but it does not directly test why those patterns occurred. Future One Health analyses should link human surveillance data with veterinary, environmental, and agricultural indicators to examine whether livestock density, animal infection prevalence, vaccination coverage, climatic variability, vegetation phenology, and animal movement explain the observed county-level differences. The occupational distribution reported here reflects case composition rather than occupation-specific incidence, because occupation-stratified denominator data were not available. Farmers and herders accounted for 77.3% of reported cases (9,448 of 12,220); the median age of all reported cases was 47.0 years (IQR 37.0–54.0). Estimating true excess risk by occupation would require occupation- and age-specific population denominators or linked labor-force exposure data.

### 4.4 Public health implications

For prevention planning, the clearest implication is that preparedness should start before March. Occupational protection campaigns, diagnostic training in primary care, and laboratory readiness need to be in place before the March–July high-incidence period. This timing is anchored to the observed rise in human cases from March and is compatible with intensified livestock handling around spring lambing and calving; exact animal-vaccination calendars were not available in this human-surveillance dataset and should be aligned with local veterinary schedules.

Spatial targeting should keep workload and relative excess in view at the same time. Zhalantun City, Arun Banner, and Morin Dawa Daur Autonomous Banner remain important for service capacity and case management because they contributed many cases. New Barag Right Banner, New Barag Left Banner, and Chen Barag Banner require targeted surveillance review and risk investigation because their denominator-linked indicators were high. Prevention should also remain centered on farmers, herders, animal-product handlers, household contacts, and other occupationally exposed groups.

### 4.5 Strengths, limitations, and future directions

This study has several strengths. It used a decade of city-wide surveillance data, examined patterns at the county level, incorporated county-year population denominators for 2016–2024, and combined descriptive epidemiology with population-adjusted incidence estimation, scan analysis, hotspot exploration, Bayesian smoothing, forecasting, and disability burden estimation. The updated population-denominator analysis improves the epidemiological interpretation of spatial heterogeneity and directly addresses a key limitation of analyses based only on surveillance case counts.

Several limitations should be considered. NNDRS-based surveillance data are subject to underdiagnosis and underreporting, particularly for mild, atypical, or delayed cases. County-level population denominators were formally used only through 2024, so denominator-based spatial scan analysis, county incidence ranking, and BYM offset modeling were restricted to 2016–2024 and could not formally estimate denominator-based risk for 2025. The 2025 deletion rate (37.5%) was substantially higher than the 2016–2024 range (8.4–26.3%), and retained-versus-deleted comparisons could not prove full analytic comparability; 2025 findings should therefore be interpreted as provisional reported-case evidence. The denominator itself was based on household-registered population rather than resident or occupation-specific exposed population. Although a 10–30% denominator-inflation sensitivity analysis was conducted for selected pastoral or high-mobility counties, the true exposed population remains unknown, and fine ranking among high-incidence counties may change under plausible denominator inflation.

The analysis was also limited by the absence of individual-level exposure information, animal brucellosis surveillance data, livestock movement records, livestock density, livestock vaccination coverage, climate variables, NDVI, land use, and socioeconomic covariates. The observed spatiotemporal patterns therefore should not be read as direct evidence of specific transmission mechanisms. Model-specific caveats also remain: the BYM results are probabilistic small-area smoothing estimates from only 13 counties, DALY estimates depend on literature-based disability weights and assumed duration rather than local follow-up data, and short-term forecasts are based on a relatively short monthly series with incomplete prediction-interval calibration and potential recursive error accumulation for LightGBM.

Future studies should link human surveillance data with veterinary, environmental, agricultural, and socioeconomic information to better explain transmission dynamics, evaluate specific interventions, and refine risk prediction. If monthly climate, vegetation, livestock, and animal-infection indicators become available, future models could examine lagged climate-sensitive zoonotic-disease dynamics and compare purely temporal forecasts with covariate-informed early-warning models.

## 5 Conclusion

Human brucellosis in Hulunbuir remained endemic during 2016–2025, with clear seasonality and marked county-level heterogeneity. The denominator-linked 2016–2024 panel provided the strongest basis for formal spatial interpretation, while 2025 findings remained provisional. Monte Carlo-supported scan windows and BYM smoothing highlighted counties requiring surveillance review, and forecasting may help local teams prepare before the March–July high-incidence period. Approximate non-fatal burden estimates were sensitive to disability-weight and duration assumptions, underscoring the need for local follow-up data. Future One Health-oriented studies should integrate human, animal, environmental, and occupational exposure data to explain the mechanisms underlying the observed surveillance patterns.

## Data Availability

The original contributions presented in the study are included in the article/Supplementary material, further inquiries can be directed to the corresponding author.
