# Supplementary material for: County-level spatiotemporal patterns, forecasting, and non-fatal burden of human brucellosis in Hulunbuir, China, 2016–2025
Source: Front Public Health. 2026 Jul 9;14:1876534. doi: 10.3389/fpubh.2026.1876534 (PMC13391891; doi:10.3389/fpubh.2026.1876534)
Supplement: Supplementary file 1 [file Supplementary_file_1.DOCX]

# Supplementary Tables S1–S16

## Supplementary Table S1. Annual data cleaning flow

The 2025 deletion rate was substantially higher than the 2016–2024 range; 2025 analyses were treated as provisional reported-case evidence.

| Year | Original records | Deleted records | Included records | Deletion rate (%) | Notes |
| --- | --- | --- | --- | --- | --- |
| 2016 | 815 | 71 | 744 | 8.7 |  |
| 2017 | 922 | 113 | 809 | 12.3 |  |
| 2018 | 996 | 143 | 853 | 14.4 |  |
| 2019 | 1,681 | 141 | 1,540 | 8.4 |  |
| 2020 | 1,364 | 115 | 1,249 | 8.4 |  |
| 2021 | 1,842 | 178 | 1,664 | 9.7 |  |
| 2022 | 1,663 | 286 | 1,377 | 17.2 |  |
| 2023 | 1,704 | 304 | 1,400 | 17.8 |  |
| 2024 | 1,613 | 425 | 1,188 | 26.3 | Highest pre-2025 deletion rate |
| 2025 | 2,235 | 839 | 1,396 | 37.5 | Provisional; high deletion rate |

## Supplementary Table S2. Comparison between retained and deleted records in 2025

Note: Deleted cards had not passed final audit and may include duplicate or non-analytic records; therefore, this comparison describes available raw-file differences but does not establish analytic comparability for final variables.

| Variable | Category | Retained 2025 records n (%) | Deleted 2025 records n (%) | p-value | Cramer's V |
| --- | --- | --- | --- | --- | --- |
| County | Arun Banner | 209 (15.0%) | 203 (24.2%) | <0.001 | 0.349 |
|  | Chen Barag Banner | 110 (7.9%) | 28 (3.3%) |  |  |
|  | Erguna City | 72 (5.2%) | 51 (6.1%) |  |  |
|  | Ewenki Autonomous Banner | 112 (8.0%) | 44 (5.2%) |  |  |
|  | Genhe City | 7 (0.5%) | 0 (0.0%) |  |  |
|  | Hailar District | 115 (8.2%) | 17 (2.0%) |  |  |
|  | Manzhouli City | 13 (0.9%) | 9 (1.1%) |  |  |
|  | Morin Dawa Daur Autonomous Banner | 224 (16.0%) | 36 (4.3%) |  |  |
|  | New Barag Left Banner | 55 (3.9%) | 77 (9.2%) |  |  |
|  | New Barag Right Banner | 98 (7.0%) | 46 (5.5%) |  |  |
|  | Oroqen Autonomous Banner | 95 (6.8%) | 35 (4.2%) |  |  |
|  | Yakeshi City | 48 (3.4%) | 3 (0.4%) |  |  |
|  | Zhalantun City | 238 (17.0%) | 290 (34.6%) |  |  |
| Onset month | 1 | 137 (9.8%) | 35 (4.2%) | <0.001 | 0.165 |
|  | 2 | 130 (9.3%) | 50 (6.0%) |  |  |
|  | 3 | 153 (11.0%) | 84 (10.0%) |  |  |
|  | 4 | 146 (10.5%) | 61 (7.3%) |  |  |
|  | 5 | 123 (8.8%) | 97 (11.6%) |  |  |
|  | 6 | 146 (10.5%) | 119 (14.2%) |  |  |
|  | 7 | 103 (7.4%) | 103 (12.3%) |  |  |
|  | 8 | 112 (8.0%) | 79 (9.4%) |  |  |
|  | 9 | 87 (6.2%) | 49 (5.8%) |  |  |
|  | 10 | 71 (5.1%) | 45 (5.4%) |  |  |
|  | 11 | 82 (5.9%) | 54 (6.4%) |  |  |
|  | 12 | 106 (7.6%) | 63 (7.5%) |  |  |
| Sex | Female | 488 (35.0%) | 286 (34.1%) | 0.710 | 0.008 |
|  | Male | 908 (65.0%) | 553 (65.9%) |  |  |
| Age group | 20–39 | 316 (22.6%) | 207 (24.7%) | 0.003 | 0.080 |
|  | 40–59 | 836 (59.9%) | 491 (58.5%) |  |  |
|  | <20 | 24 (1.7%) | 33 (3.9%) |  |  |
|  | >=60 | 220 (15.8%) | 108 (12.9%) |  |  |
| Occupation | Farmer | 822 (58.9%) | 514 (61.3%) | <0.001 | 0.115 |
|  | Herder | 269 (19.3%) | 130 (15.5%) |  |  |
|  | Household/unemployed | 168 (12.0%) | 86 (10.3%) |  |  |
|  | Other/unknown | 2 (0.1%) | 6 (0.7%) |  |  |
|  | Retired | 32 (2.3%) | 18 (2.1%) |  |  |
|  | Service/food/business | 16 (1.1%) | 4 (0.5%) |  |  |
|  | Staff/cadre/professional | 34 (2.4%) | 17 (2.0%) |  |  |
|  | Student/child | 21 (1.5%) | 31 (3.7%) |  |  |
|  | Worker | 32 (2.3%) | 33 (3.9%) |  |  |
| Case classification | Clinically diagnosed case | 334 (23.9%) | 279 (33.3%) | <0.001 | 0.113 |
|  | Suspected case | 0 (0.0%) | 3 (0.4%) |  |  |
|  | Confirmed case | 1062 (76.1%) | 557 (66.4%) |  |  |
| Reporting unit type | A100 | 582 (41.7%) | 426 (50.8%) | <0.001 | 0.126 |
|  | A210 | 84 (6.0%) | 41 (4.9%) |  |  |
|  | A411 | 332 (23.8%) | 203 (24.2%) |  |  |
|  | A516 | 1 (0.1%) | 0 (0.0%) |  |  |
|  | A519 | 0 (0.0%) | 1 (0.1%) |  |  |
|  | A521 | 13 (0.9%) | 9 (1.1%) |  |  |
|  | B100 | 3 (0.2%) | 1 (0.1%) |  |  |
|  | C210 | 4 (0.3%) | 1 (0.1%) |  |  |
|  | C220 | 4 (0.3%) | 0 (0.0%) |  |  |
|  | D110 | 93 (6.7%) | 33 (3.9%) |  |  |
|  | J100 | 280 (20.1%) | 122 (14.5%) |  |  |
|  | M500 | 0 (0.0%) | 1 (0.1%) |  |  |
|  | M626 | 0 (0.0%) | 1 (0.1%) |  |  |

## Supplementary Table S3. DALY sensitivity analysis including and excluding 2025

YLL was set to zero because no valid death records were available in the cleaned surveillance table and no independent mortality linkage was available.

| Period | Included cases | DW | Duration | Estimated YLD/DALY | Interpretation |
| --- | --- | --- | --- | --- | --- |
| 2016–2024 | 10,824 | 0.25 | 0.5 years | 1,353.0 | Excluding provisional 2025 |
| 2016–2025 | 12,220 | 0.25 | 0.5 years | 1,527.5 | Including provisional 2025; upper-bound proxy |

## Supplementary Table S4. DALY two-way sensitivity analysis by disability weight and duration

Values use 12,220 reported cases from 2016–2025, including provisional 2025 records; results are YLD-based approximate non-fatal burden estimates. The corresponding 2016–2024 estimate under the 6-month, DW = 0.250 scenario is provided in Supplementary Table S3.

| DW scenario | DW | Duration 3 months | Duration 6 months | Duration 12 months |
| --- | --- | --- | --- | --- |
| Brucellosis-specific chronic localized | 0.15 | 458.2 | 916.5 | 1,833.0 |
| Brucellosis-specific acute | 0.19 | 580.5 | 1,160.9 | 2,321.8 |
| GBD 2021 post-acute proxy | 0.219 | 669.0 | 1,338.1 | 2,676.2 |
| GBD 2010 post-acute upper-bound proxy | 0.25 | 763.8 | 1,527.5 | 3,055.0 |

## Supplementary Table S5. Population denominator sensitivity analysis

Selected pastoral or high-mobility counties were inflated by 10%, 20%, and 30% to assess potential household-registration denominator bias.

| Scenario | Adjusted counties | Top 1 | Top 2 | Top 3 | Top 4 | Top 5 | Ranking change |
| --- | --- | --- | --- | --- | --- | --- | --- |
| Original denominator | None | New Barag Right Banner | New Barag Left Banner | Chen Barag Banner | Arun Banner | Zhalantun City | Reference |
| +10% exposed population | New Barag Right Banner; New Barag Left Banner; Chen Barag Banner; Ewenki Autonomous Banner; Erguna City | New Barag Right Banner | New Barag Left Banner | Arun Banner | Chen Barag Banner | Zhalantun City | Top-five order changed; New Barag Right Banner remained first |
| +20% exposed population | New Barag Right Banner; New Barag Left Banner; Chen Barag Banner; Ewenki Autonomous Banner; Erguna City | New Barag Right Banner | Arun Banner | New Barag Left Banner | Zhalantun City | Chen Barag Banner | Top-five order changed; New Barag Right Banner remained first |
| +30% exposed population | New Barag Right Banner; New Barag Left Banner; Chen Barag Banner; Ewenki Autonomous Banner; Erguna City | New Barag Right Banner | Arun Banner | Zhalantun City | New Barag Left Banner | Chen Barag Banner | Top-five order changed; New Barag Right Banner remained first |

## Supplementary Table S6. BYM posterior uncertainty outputs

County-level summaries were computed from posterior draws by averaging county-year RR draws across 2016–2024 for each county.

| County | Posterior mean RR | Posterior median RR | 95% CrI lower | 95% CrI upper | P(RR > 1) | P(RR > 1.5) |
| --- | --- | --- | --- | --- | --- | --- |
| New Barag Right Banner | 3.209 | 3.206 | 2.935 | 3.493 | 1.000 | 1.000 |
| New Barag Left Banner | 1.891 | 1.888 | 1.694 | 2.094 | 1.000 | 1.000 |
| Chen Barag Banner | 1.773 | 1.772 | 1.607 | 1.948 | 1.000 | 1.000 |
| Arun Banner | 1.622 | 1.621 | 1.555 | 1.689 | 1.000 | 1.000 |
| Zhalantun City | 1.531 | 1.531 | 1.473 | 1.590 | 1.000 | 0.850 |
| Erguna City | 1.488 | 1.487 | 1.364 | 1.620 | 1.000 | 0.415 |
| Ewenki Autonomous Banner | 1.043 | 1.043 | 0.962 | 1.125 | 0.849 | 0.000 |
| Morin Dawa Daur Autonomous Banner | 0.997 | 0.997 | 0.945 | 1.049 | 0.454 | 0.000 |
| Oroqen Autonomous Banner | 0.607 | 0.607 | 0.561 | 0.654 | 0.000 | 0.000 |
| Hailar District | 0.550 | 0.550 | 0.510 | 0.592 | 0.000 | 0.000 |
| Yakeshi City | 0.372 | 0.372 | 0.340 | 0.405 | 0.000 | 0.000 |
| Manzhouli City | 0.227 | 0.226 | 0.182 | 0.278 | 0.000 | 0.000 |
| Genhe City | 0.044 | 0.043 | 0.028 | 0.063 | 0.000 | 0.000 |

## Supplementary Table S7. BYM sensitivity analysis by spatial rho and adjacency

County-level RR summaries average county-year posterior RR draws across 2016–2024 for each county. Rhat and ESS are reported as maximum Rhat and minimum ESS across monitored scalar, county, and year parameters. Queen and rook adjacency produced identical county-neighbor structures in the available GeoJSON; therefore, the queen-adjacency sensitivity row is numerically identical to the rho = 0.99 rook-adjacency row.

| Model setting | rho | Adjacency | Top-five counties by median RR | New Barag Right Banner median RR | New Barag Right Banner 95% CrI | New Barag Right Banner P(RR > 1.5) | Max Rhat | Min bulk ESS | Min tail ESS | Divergences | Max-tree-depth hits |
| --- | --- | --- | --- | --- | --- | --- | --- | --- | --- | --- | --- |
| Rook adjacency, rho = 0.5 | 0.5 | Rook | New Barag Right Banner; New Barag Left Banner; Chen Barag Banner; Arun Banner; Zhalantun City | 3.208 | 2.936–3.502 | 1.000 | 1.000 | 1385 | 3020 | 0 | 0 |
| Rook adjacency, rho = 0.8 | 0.8 | Rook | New Barag Right Banner; New Barag Left Banner; Chen Barag Banner; Arun Banner; Zhalantun City | 3.207 | 2.933–3.496 | 1.000 | 1.000 | 1297 | 2281 | 0 | 0 |
| Rook adjacency, rho = 0.95 | 0.95 | Rook | New Barag Right Banner; New Barag Left Banner; Chen Barag Banner; Arun Banner; Zhalantun City | 3.210 | 2.929–3.502 | 1.000 | 1.000 | 1391 | 1807 | 0 | 0 |
| Rook adjacency, rho = 0.99 | 0.99 | Rook | New Barag Right Banner; New Barag Left Banner; Chen Barag Banner; Arun Banner; Zhalantun City | 3.206 | 2.935–3.493 | 1.000 | 1.000 | 2208 | 1580 | 0 | 0 |
| Queen adjacency, rho = 0.99 | 0.99 | Queen | New Barag Right Banner; New Barag Left Banner; Chen Barag Banner; Arun Banner; Zhalantun City | 3.206 | 2.935–3.493 | 1.000 | 1.000 | 2208 | 1580 | 0 | 0 |

## Supplementary Table S8. Decision-support interpretation of integrated framework

| Analytic component | Output | Decision-support value | Single-method limitation addressed |
| --- | --- | --- | --- |
| Absolute case counts | High service-burden counties | Allocate diagnostic and case-management capacity to high-volume counties (Zhalantun City, Arun Banner, Morin Dawa Daur) | Raw counts may favor large-population counties |
| Population-adjusted incidence | High relative-risk counties | Prioritize targeted surveillance review in small high-relative-risk counties (New Barag Right/Left Banner, Chen Barag Banner) | Incidence alone may miss service workload |
| O/E excess and LLR-ranked windows | County-year excess signals | Flag county-year excess windows (e.g., Arun Banner 2019) for retrospective outbreak/reporting review | Static maps cannot show short-window excess |
| BYM smoothing | Stabilized relative-risk estimates with uncertainty | Reduce small-area random fluctuation | Crude rates are unstable in small counties |
| Seasonality and forecasting | Pre-season pressure estimate | Prepare before March–July high-incidence period | Descriptive annual totals do not support preparedness timing |
| DALY estimation | Approximate non-fatal burden | Communicate disease impact beyond case counts | Case counts do not reflect disability burden |

## Supplementary Table S9. Provisional 2025 forecasting benchmark with additional exploratory models.

The 2025 benchmark used provisional records and should not be interpreted as primary evidence of model superiority.

| Model | MAE | RMSE | MAPE (%) | Interpretation |
| --- | --- | --- | --- | --- |
| SARIMA | 21.92 | 27.51 | 24.81 | 2025 provisional benchmark |
| Prophet | 23.18 | 28.02 | 27.76 | 2025 provisional benchmark |
| Holt-Winters | 33.29 | 38.41 | 41.38 | Exploratory 2025 provisional benchmark |
| LightGBM | 22.00 | 27.57 | 21.67 | 2025 provisional benchmark |
| Poisson GLM | 46.06 | 51.34 | 51.54 | Exploratory 2025 provisional benchmark |
| FITS | 28.10 | 33.94 | 30.04 | Exploratory 2025 provisional benchmark |
| TimesFM | 22.23 | 26.17 | 29.67 | Exploratory 2025 provisional benchmark |
| Chronos-2 | 36.61 | 43.66 | 38.41 | Exploratory 2025 provisional benchmark |

Supplementary Table S10. Comparison between previous proportional and revised population-based expected-count specifications for scan-statistic window ranking.

| Expected-count specification | Window type | Rank | County | Period | O | E | RR/OE | LLR | Monte Carlo p-value | Interpretation note |
| --- | --- | --- | --- | --- | --- | --- | --- | --- | --- | --- |
| Previous proportional | Single-year | 1 | Arun Banner | 2019 | 466 | 327.38 | 1.42 | 25.91 | NA | Compares observed cases with the county's long-term reported-case contribution. |
| Previous proportional | Single-year | 2 | Hailar District | 2016 | 86 | 48.46 | 1.77 | 11.79 | NA | Compares observed cases with the county's long-term reported-case contribution. |
| Previous proportional | Single-year | 3 | Zhalantun City | 2021 | 521 | 419.07 | 1.24 | 11.50 | NA | Compares observed cases with the county's long-term reported-case contribution. |
| Previous proportional | Single-year | 4 | Zhalantun City | 2017 | 257 | 203.74 | 1.26 | 6.42 | NA | Compares observed cases with the county's long-term reported-case contribution. |
| Previous proportional | Single-year | 5 | New Barag Left Banner | 2016 | 43 | 24.13 | 1.78 | 5.98 | NA | Compares observed cases with the county's long-term reported-case contribution. |
| Previous proportional | Two-year | 1 | Arun Banner | 2018–2019 | 695 | 508.71 | 1.37 | 30.57 | NA | Compares observed cases with the county's long-term reported-case contribution. |
| Previous proportional | Two-year | 2 | Arun Banner | 2019–2020 | 764 | 592.89 | 1.29 | 22.61 | NA | Compares observed cases with the county's long-term reported-case contribution. |
| Previous proportional | Two-year | 3 | Zhalantun City | 2017–2018 | 519 | 418.57 | 1.24 | 11.19 | NA | Compares observed cases with the county's long-term reported-case contribution. |
| Previous proportional | Two-year | 4 | New Barag Left Banner | 2016–2017 | 84 | 50.36 | 1.67 | 9.34 | NA | Compares observed cases with the county's long-term reported-case contribution. |
| Previous proportional | Two-year | 5 | Zhalantun City | 2020–2021 | 846 | 733.63 | 1.15 | 8.20 | NA | Compares observed cases with the county's long-term reported-case contribution. |
| Revised population-based | Single-year | 1 | Arun Banner | 2019 | 466 | 200.74 | 2.32 | 127.20 | <0.001 | Main analysis; compares observed cases with county-year population share. |
| Revised population-based | Single-year | 2 | Zhalantun City | 2021 | 521 | 273.76 | 1.90 | 88.03 | <0.001 | Main analysis; compares observed cases with county-year population share. |
| Revised population-based | Single-year | 3 | New Barag Right Banner | 2022 | 85 | 20.13 | 4.22 | 57.56 | <0.001 | Main analysis; compares observed cases with county-year population share. |
| Revised population-based | Single-year | 4 | Arun Banner | 2018 | 229 | 110.61 | 2.07 | 48.26 | <0.001 | Main analysis; compares observed cases with county-year population share. |
| Revised population-based | Single-year | 5 | Zhalantun City | 2017 | 257 | 132.75 | 1.94 | 45.52 | <0.001 | Main analysis; compares observed cases with county-year population share. |
| Revised population-based | Two-year | 1 | Arun Banner | 2018–2019 | 695 | 311.11 | 2.23 | 174.73 | <0.001 | Main analysis; compares observed cases with county-year population share. |
| Revised population-based | Two-year | 2 | Arun Banner | 2019–2020 | 764 | 364.58 | 2.10 | 165.81 | <0.001 | Main analysis; compares observed cases with county-year population share. |
| Revised population-based | Two-year | 3 | Zhalantun City | 2020–2021 | 846 | 479.22 | 1.77 | 114.05 | <0.001 | Main analysis; compares observed cases with county-year population share. |
| Revised population-based | Two-year | 4 | New Barag Right Banner | 2022–2023 | 159 | 40.76 | 3.90 | 98.18 | <0.001 | Main analysis; compares observed cases with county-year population share. |
| Revised population-based | Two-year | 5 | Zhalantun City | 2021–2022 | 833 | 500.44 | 1.66 | 91.89 | <0.001 | Main analysis; compares observed cases with county-year population share. |

Note: Previous proportional expected-count approach evaluates whether a county-year exceeds its own long-term reported-case contribution.

Note: Revised population-based expected-count approach evaluates whether observed cases exceed what would be expected from county-year population size.

Note: Monte Carlo p-values are reported for the revised population-based scan only.

Supplementary Table S11. Full annual local Getis-Ord Gi* outputs under rook adjacency, 2016–2024.

| County | Year | Cases | Population | Incidence per 100,000 | Gi* Z | Upper-tail p-value | BH-FDR q-value | Uncorrected p < 0.05 | FDR q < 0.05 |
| --- | --- | --- | --- | --- | --- | --- | --- | --- | --- |
| Zhalantun City | 2016 | 134 | 412011 | 32.52 | -0.74 | 0.772 | 0.963 | No | No |
| New Barag Right Banner | 2016 | 27 | 35138 | 76.84 | 1.60 | 0.054 | 0.708 | No | No |
| New Barag Left Banner | 2016 | 43 | 42093 | 102.15 | 1.66 | 0.049 | 0.708 | Yes | No |
| Genhe City | 2016 | 2 | 140056 | 1.43 | -1.26 | 0.896 | 0.963 | No | No |
| Hailar District | 2016 | 86 | 282726 | 30.42 | -0.36 | 0.640 | 0.963 | No | No |
| Manzhouli City | 2016 | 4 | 84488 | 4.73 | 1.60 | 0.054 | 0.708 | No | No |
| Yakeshi City | 2016 | 53 | 335827 | 15.78 | -1.23 | 0.890 | 0.963 | No | No |
| Morin Dawa Daur Autonomous Banner | 2016 | 86 | 319345 | 26.93 | -0.52 | 0.697 | 0.963 | No | No |
| Oroqen Autonomous Banner | 2016 | 65 | 254566 | 25.53 | -1.64 | 0.949 | 0.963 | No | No |
| Ewenki Autonomous Banner | 2016 | 41 | 139403 | 29.41 | 0.81 | 0.209 | 0.848 | No | No |
| Arun Banner | 2016 | 128 | 320766 | 39.90 | -1.01 | 0.844 | 0.963 | No | No |
| Chen Barag Banner | 2016 | 34 | 56400 | 60.28 | 1.17 | 0.122 | 0.777 | No | No |
| Erguna City | 2016 | 41 | 80991 | 50.62 | -0.52 | 0.699 | 0.963 | No | No |
| Zhalantun City | 2017 | 257 | 409048 | 62.83 | -0.19 | 0.575 | 0.963 | No | No |
| New Barag Right Banner | 2017 | 37 | 35190 | 105.14 | 1.75 | 0.040 | 0.708 | Yes | No |
| New Barag Left Banner | 2017 | 41 | 42156 | 97.26 | 1.73 | 0.042 | 0.708 | Yes | No |
| Genhe City | 2017 | 4 | 137269 | 2.91 | -1.68 | 0.953 | 0.963 | No | No |
| Hailar District | 2017 | 48 | 284434 | 16.88 | -0.64 | 0.738 | 0.963 | No | No |
| Manzhouli City | 2017 | 5 | 85311 | 5.86 | 1.75 | 0.040 | 0.708 | Yes | No |
| Yakeshi City | 2017 | 47 | 331099 | 14.20 | -1.35 | 0.911 | 0.963 | No | No |
| Morin Dawa Daur Autonomous Banner | 2017 | 82 | 320320 | 25.60 | -0.59 | 0.722 | 0.963 | No | No |
| Oroqen Autonomous Banner | 2017 | 46 | 252113 | 18.25 | -1.57 | 0.942 | 0.963 | No | No |
| Ewenki Autonomous Banner | 2017 | 36 | 138672 | 25.96 | 0.75 | 0.227 | 0.885 | No | No |
| Arun Banner | 2017 | 145 | 320562 | 45.23 | -0.55 | 0.709 | 0.963 | No | No |
| Chen Barag Banner | 2017 | 37 | 55955 | 66.12 | 0.20 | 0.421 | 0.963 | No | No |
| Erguna City | 2017 | 24 | 80640 | 29.76 | -0.82 | 0.794 | 0.963 | No | No |
| Zhalantun City | 2018 | 262 | 406596 | 64.44 | 0.60 | 0.275 | 0.963 | No | No |
| New Barag Right Banner | 2018 | 37 | 35223 | 105.04 | 0.96 | 0.168 | 0.800 | No | No |
| New Barag Left Banner | 2018 | 17 | 41991 | 40.48 | 0.81 | 0.210 | 0.848 | No | No |
| Genhe City | 2018 | 4 | 133711 | 2.99 | -1.34 | 0.909 | 0.963 | No | No |
| Hailar District | 2018 | 42 | 285654 | 14.70 | -1.03 | 0.848 | 0.963 | No | No |
| Manzhouli City | 2018 | 9 | 86753 | 10.37 | 0.96 | 0.168 | 0.800 | No | No |
| Yakeshi City | 2018 | 33 | 325815 | 10.13 | -0.60 | 0.724 | 0.963 | No | No |
| Morin Dawa Daur Autonomous Banner | 2018 | 74 | 319569 | 23.16 | -0.09 | 0.538 | 0.963 | No | No |
| Oroqen Autonomous Banner | 2018 | 33 | 248462 | 13.28 | -1.25 | 0.895 | 0.963 | No | No |
| Ewenki Autonomous Banner | 2018 | 46 | 137863 | 33.37 | -0.39 | 0.652 | 0.963 | No | No |
| Arun Banner | 2018 | 229 | 321339 | 71.26 | -0.08 | 0.533 | 0.963 | No | No |
| Chen Barag Banner | 2018 | 22 | 55239 | 39.83 | -0.54 | 0.706 | 0.963 | No | No |
| Erguna City | 2018 | 45 | 79942 | 56.29 | -0.80 | 0.789 | 0.963 | No | No |
| Zhalantun City | 2019 | 395 | 404272 | 97.71 | 0.67 | 0.252 | 0.951 | No | No |
| New Barag Right Banner | 2019 | 57 | 35180 | 162.02 | 1.06 | 0.144 | 0.800 | No | No |
| New Barag Left Banner | 2019 | 45 | 41813 | 107.62 | 0.90 | 0.185 | 0.801 | No | No |
| Genhe City | 2019 | 2 | 130722 | 1.53 | -1.73 | 0.958 | 0.963 | No | No |
| Hailar District | 2019 | 78 | 287405 | 27.14 | -1.04 | 0.851 | 0.963 | No | No |
| Manzhouli City | 2019 | 11 | 87861 | 12.52 | 1.06 | 0.144 | 0.800 | No | No |
| Yakeshi City | 2019 | 60 | 321174 | 18.68 | -0.92 | 0.822 | 0.963 | No | No |
| Morin Dawa Daur Autonomous Banner | 2019 | 198 | 316398 | 62.58 | 0.33 | 0.372 | 0.963 | No | No |
| Oroqen Autonomous Banner | 2019 | 42 | 244979 | 17.14 | -0.97 | 0.833 | 0.963 | No | No |
| Ewenki Autonomous Banner | 2019 | 84 | 136932 | 61.34 | -0.18 | 0.572 | 0.963 | No | No |
| Arun Banner | 2019 | 466 | 320821 | 145.25 | 0.10 | 0.460 | 0.963 | No | No |
| Chen Barag Banner | 2019 | 38 | 54551 | 69.66 | -0.36 | 0.642 | 0.963 | No | No |
| Erguna City | 2019 | 64 | 79155 | 80.85 | -1.11 | 0.867 | 0.963 | No | No |
| Zhalantun City | 2020 | 325 | 401271 | 80.99 | 0.41 | 0.341 | 0.963 | No | No |
| New Barag Right Banner | 2020 | 60 | 35012 | 171.37 | 1.25 | 0.105 | 0.766 | No | No |
| New Barag Left Banner | 2020 | 32 | 41431 | 77.24 | 1.39 | 0.082 | 0.739 | No | No |
| Genhe City | 2020 | 1 | 127217 | 0.79 | -1.79 | 0.963 | 0.963 | No | No |
| Hailar District | 2020 | 66 | 288256 | 22.90 | -0.65 | 0.743 | 0.963 | No | No |
| Manzhouli City | 2020 | 8 | 88239 | 9.07 | 1.25 | 0.105 | 0.766 | No | No |
| Yakeshi City | 2020 | 54 | 315130 | 17.14 | -1.12 | 0.870 | 0.963 | No | No |
| Morin Dawa Daur Autonomous Banner | 2020 | 174 | 314218 | 55.38 | 0.06 | 0.475 | 0.963 | No | No |
| Oroqen Autonomous Banner | 2020 | 64 | 240728 | 26.59 | -1.15 | 0.874 | 0.963 | No | No |
| Ewenki Autonomous Banner | 2020 | 92 | 136292 | 67.50 | -0.06 | 0.525 | 0.963 | No | No |
| Arun Banner | 2020 | 298 | 319778 | 93.19 | -0.14 | 0.557 | 0.963 | No | No |
| Chen Barag Banner | 2020 | 38 | 53759 | 70.69 | -0.47 | 0.682 | 0.963 | No | No |
| Erguna City | 2020 | 37 | 77947 | 47.47 | -1.21 | 0.887 | 0.963 | No | No |
| Zhalantun City | 2021 | 521 | 396980 | 131.24 | 0.17 | 0.432 | 0.963 | No | No |
| New Barag Right Banner | 2021 | 68 | 35090 | 193.79 | 0.92 | 0.178 | 0.800 | No | No |
| New Barag Left Banner | 2021 | 37 | 41467 | 89.23 | 0.97 | 0.166 | 0.800 | No | No |
| Genhe City | 2021 | 2 | 123784 | 1.62 | -1.26 | 0.895 | 0.963 | No | No |
| Hailar District | 2021 | 75 | 288617 | 25.99 | -0.88 | 0.812 | 0.963 | No | No |
| Manzhouli City | 2021 | 16 | 88799 | 18.02 | 0.92 | 0.178 | 0.800 | No | No |
| Yakeshi City | 2021 | 94 | 308989 | 30.42 | -0.82 | 0.795 | 0.963 | No | No |
| Morin Dawa Daur Autonomous Banner | 2021 | 228 | 309919 | 73.57 | -0.21 | 0.585 | 0.963 | No | No |
| Oroqen Autonomous Banner | 2021 | 103 | 235331 | 43.77 | -1.45 | 0.927 | 0.963 | No | No |
| Ewenki Autonomous Banner | 2021 | 86 | 135482 | 63.48 | -0.09 | 0.536 | 0.963 | No | No |
| Arun Banner | 2021 | 291 | 318133 | 91.47 | -0.07 | 0.527 | 0.963 | No | No |
| Chen Barag Banner | 2021 | 55 | 53338 | 103.12 | -0.27 | 0.606 | 0.963 | No | No |
| Erguna City | 2021 | 88 | 77078 | 114.17 | -0.59 | 0.721 | 0.963 | No | No |
| Zhalantun City | 2022 | 312 | 393846 | 79.22 | -0.45 | 0.673 | 0.963 | No | No |
| New Barag Right Banner | 2022 | 85 | 34979 | 243.00 | 1.14 | 0.126 | 0.777 | No | No |
| New Barag Left Banner | 2022 | 29 | 41299 | 70.22 | 1.32 | 0.093 | 0.766 | No | No |
| Genhe City | 2022 | 2 | 120942 | 1.65 | -1.12 | 0.869 | 0.963 | No | No |
| Hailar District | 2022 | 109 | 288505 | 37.78 | -0.50 | 0.692 | 0.963 | No | No |
| Manzhouli City | 2022 | 12 | 88381 | 13.58 | 1.14 | 0.126 | 0.777 | No | No |
| Yakeshi City | 2022 | 55 | 304020 | 18.09 | -0.91 | 0.820 | 0.963 | No | No |
| Morin Dawa Daur Autonomous Banner | 2022 | 181 | 308202 | 58.73 | -0.36 | 0.641 | 0.963 | No | No |
| Oroqen Autonomous Banner | 2022 | 89 | 231506 | 38.44 | -1.45 | 0.926 | 0.963 | No | No |
| Ewenki Autonomous Banner | 2022 | 81 | 135072 | 59.97 | -0.42 | 0.664 | 0.963 | No | No |
| Arun Banner | 2022 | 271 | 316772 | 85.55 | -0.74 | 0.772 | 0.963 | No | No |
| Chen Barag Banner | 2022 | 64 | 52741 | 121.35 | -0.12 | 0.546 | 0.963 | No | No |
| Erguna City | 2022 | 87 | 76259 | 114.08 | -0.33 | 0.629 | 0.963 | No | No |
| Zhalantun City | 2023 | 275 | 390104 | 70.49 | -0.66 | 0.745 | 0.963 | No | No |
| New Barag Right Banner | 2023 | 74 | 34874 | 212.19 | 1.49 | 0.068 | 0.708 | No | No |
| New Barag Left Banner | 2023 | 54 | 41106 | 131.37 | 1.79 | 0.037 | 0.708 | Yes | No |
| Genhe City | 2023 | 2 | 117613 | 1.70 | -1.43 | 0.923 | 0.963 | No | No |
| Hailar District | 2023 | 116 | 287641 | 40.33 | -0.39 | 0.651 | 0.963 | No | No |
| Manzhouli City | 2023 | 12 | 88842 | 13.51 | 1.49 | 0.068 | 0.708 | No | No |
| Yakeshi City | 2023 | 70 | 297940 | 23.49 | -1.31 | 0.906 | 0.963 | No | No |
| Morin Dawa Daur Autonomous Banner | 2023 | 218 | 305998 | 71.24 | -0.37 | 0.645 | 0.963 | No | No |
| Oroqen Autonomous Banner | 2023 | 106 | 227155 | 46.66 | -1.57 | 0.942 | 0.963 | No | No |
| Ewenki Autonomous Banner | 2023 | 93 | 134790 | 69.00 | 0.13 | 0.449 | 0.963 | No | No |
| Arun Banner | 2023 | 240 | 313062 | 76.66 | -0.89 | 0.814 | 0.963 | No | No |
| Chen Barag Banner | 2023 | 69 | 52007 | 132.67 | 0.36 | 0.359 | 0.963 | No | No |
| Erguna City | 2023 | 71 | 75192 | 94.42 | -0.53 | 0.701 | 0.963 | No | No |
| Zhalantun City | 2024 | 245 | 386908 | 63.32 | -0.62 | 0.731 | 0.963 | No | No |
| New Barag Right Banner | 2024 | 58 | 34770 | 166.81 | 1.46 | 0.073 | 0.708 | No | No |
| New Barag Left Banner | 2024 | 53 | 40705 | 130.21 | 1.91 | 0.028 | 0.708 | Yes | No |
| Genhe City | 2024 | 3 | 114812 | 2.61 | -1.45 | 0.926 | 0.963 | No | No |
| Hailar District | 2024 | 85 | 286987 | 29.62 | -0.33 | 0.628 | 0.963 | No | No |
| Manzhouli City | 2024 | 11 | 88944 | 12.37 | 1.46 | 0.073 | 0.708 | No | No |
| Yakeshi City | 2024 | 52 | 293218 | 17.73 | -1.12 | 0.869 | 0.963 | No | No |
| Morin Dawa Daur Autonomous Banner | 2024 | 148 | 303744 | 48.73 | -0.40 | 0.657 | 0.963 | No | No |
| Oroqen Autonomous Banner | 2024 | 97 | 223618 | 43.38 | -1.61 | 0.947 | 0.963 | No | No |
| Ewenki Autonomous Banner | 2024 | 76 | 134774 | 56.39 | 0.38 | 0.353 | 0.963 | No | No |
| Arun Banner | 2024 | 233 | 311133 | 74.89 | -0.93 | 0.823 | 0.963 | No | No |
| Chen Barag Banner | 2024 | 68 | 51194 | 132.83 | 0.55 | 0.291 | 0.963 | No | No |
| Erguna City | 2024 | 59 | 74888 | 78.78 | -0.38 | 0.648 | 0.963 | No | No |

Note: All 117 county-year units are retained.

Note: After Benjamini-Hochberg FDR correction across 117 county-year tests, no county-year unit reached q < 0.05; therefore, Gi* findings are exploratory.

Supplementary Table S12. Sensitivity of exploratory Gi* local spatial signals to alternative spatial-weight matrices.

| Weight matrix | County | Years with uncorrected p < 0.05 | Number of uncorrected signals | Number of FDR-significant signals | Maximum Gi* Z | Minimum upper-tail p-value | Mann-Kendall tau | Mann-Kendall p-value | Interpretation |
| --- | --- | --- | --- | --- | --- | --- | --- | --- | --- |
| Rook adjacency | Zhalantun City | None | 0 | 0 | 0.67 | 0.252 | -0.222 | 0.466 | No annual upper-tail Gi* signal at uncorrected p < 0.05. |
| KNN=3 | Ewenki Autonomous Banner | None | 0 | 0 | -0.33 | 0.628 | 0.278 | 0.348 | No annual upper-tail Gi* signal at uncorrected p < 0.05. |
| KNN=3 | Oroqen Autonomous Banner | None | 0 | 0 | -0.46 | 0.677 | 0.000 | 1.000 | No annual upper-tail Gi* signal at uncorrected p < 0.05. |
| KNN=3 | Morin Dawa Daur Autonomous Banner | None | 0 | 0 | 0.66 | 0.253 | -0.222 | 0.466 | No annual upper-tail Gi* signal at uncorrected p < 0.05. |
| KNN=3 | Yakeshi City | None | 0 | 0 | -0.52 | 0.698 | -0.333 | 0.251 | No annual upper-tail Gi* signal at uncorrected p < 0.05. |
| KNN=3 | Manzhouli City | 2016; 2017; 2023; 2024 | 4 | 0 | 2.13 | 0.017 | 0.333 | 0.251 | Exploratory uncorrected signal only; not FDR-significant. |
| KNN=3 | Hailar District | None | 0 | 0 | -0.33 | 0.628 | 0.278 | 0.348 | No annual upper-tail Gi* signal at uncorrected p < 0.05. |
| KNN=3 | Genhe City | None | 0 | 0 | -1.12 | 0.869 | 0.000 | 1.000 | No annual upper-tail Gi* signal at uncorrected p < 0.05. |
| KNN=3 | New Barag Left Banner | None | 0 | 0 | 1.35 | 0.088 | 0.000 | 1.000 | No annual upper-tail Gi* signal at uncorrected p < 0.05. |
| KNN=3 | New Barag Right Banner | 2016; 2017; 2023; 2024 | 4 | 0 | 2.13 | 0.017 | 0.333 | 0.251 | Exploratory uncorrected signal only; not FDR-significant. |
| KNN=3 | Zhalantun City | None | 0 | 0 | 0.67 | 0.252 | -0.222 | 0.466 | No annual upper-tail Gi* signal at uncorrected p < 0.05. |
| KNN=3 | Arun Banner | None | 0 | 0 | 0.68 | 0.247 | -0.222 | 0.466 | No annual upper-tail Gi* signal at uncorrected p < 0.05. |
| Inverse-distance | Zhalantun City | None | 0 | 0 | 0.50 | 0.308 | -0.222 | 0.466 | No annual upper-tail Gi* signal at uncorrected p < 0.05. |
| Inverse-distance | New Barag Left Banner | 2016; 2017; 2024 | 3 | 0 | 2.12 | 0.017 | 0.000 | 1.000 | Exploratory uncorrected signal only; not FDR-significant. |
| Inverse-distance | Genhe City | None | 0 | 0 | -0.83 | 0.796 | 0.111 | 0.754 | No annual upper-tail Gi* signal at uncorrected p < 0.05. |
| Inverse-distance | Hailar District | None | 0 | 0 | 0.85 | 0.198 | 0.278 | 0.348 | No annual upper-tail Gi* signal at uncorrected p < 0.05. |
| Inverse-distance | Manzhouli City | None | 0 | 0 | 1.53 | 0.063 | 0.222 | 0.466 | No annual upper-tail Gi* signal at uncorrected p < 0.05. |
| Inverse-distance | Yakeshi City | None | 0 | 0 | -0.87 | 0.809 | -0.389 | 0.175 | No annual upper-tail Gi* signal at uncorrected p < 0.05. |
| Inverse-distance | Morin Dawa Daur Autonomous Banner | None | 0 | 0 | -0.01 | 0.505 | 0.000 | 1.000 | No annual upper-tail Gi* signal at uncorrected p < 0.05. |
| Inverse-distance | Oroqen Autonomous Banner | None | 0 | 0 | -0.86 | 0.804 | -0.056 | 0.917 | No annual upper-tail Gi* signal at uncorrected p < 0.05. |
| Inverse-distance | Ewenki Autonomous Banner | None | 0 | 0 | 0.37 | 0.357 | 0.000 | 1.000 | No annual upper-tail Gi* signal at uncorrected p < 0.05. |
| Inverse-distance | Arun Banner | None | 0 | 0 | 0.55 | 0.290 | -0.222 | 0.466 | No annual upper-tail Gi* signal at uncorrected p < 0.05. |
| Inverse-distance | Chen Barag Banner | None | 0 | 0 | 0.22 | 0.412 | 0.111 | 0.754 | No annual upper-tail Gi* signal at uncorrected p < 0.05. |
| Inverse-distance | New Barag Right Banner | None | 0 | 0 | 1.50 | 0.067 | 0.222 | 0.466 | No annual upper-tail Gi* signal at uncorrected p < 0.05. |
| Inverse-distance | Erguna City | None | 0 | 0 | -0.52 | 0.699 | 0.000 | 1.000 | No annual upper-tail Gi* signal at uncorrected p < 0.05. |
| KNN=3 | Chen Barag Banner | None | 0 | 0 | 1.47 | 0.071 | 0.000 | 1.000 | No annual upper-tail Gi* signal at uncorrected p < 0.05. |
| Rook adjacency | Chen Barag Banner | None | 0 | 0 | 1.17 | 0.122 | 0.222 | 0.466 | No annual upper-tail Gi* signal at uncorrected p < 0.05. |
| Rook adjacency | New Barag Right Banner | 2017 | 1 | 0 | 1.75 | 0.040 | -0.056 | 0.917 | Exploratory uncorrected signal only; not FDR-significant. |
| Rook adjacency | New Barag Left Banner | 2016; 2017; 2023; 2024 | 4 | 0 | 1.91 | 0.028 | 0.333 | 0.251 | Exploratory uncorrected signal only; not FDR-significant. |
| Rook adjacency | Genhe City | None | 0 | 0 | -1.12 | 0.869 | 0.000 | 1.000 | No annual upper-tail Gi* signal at uncorrected p < 0.05. |
| Rook adjacency | Hailar District | None | 0 | 0 | -0.33 | 0.628 | 0.278 | 0.348 | No annual upper-tail Gi* signal at uncorrected p < 0.05. |
| Rook adjacency | Manzhouli City | 2017 | 1 | 0 | 1.75 | 0.040 | -0.056 | 0.917 | Exploratory uncorrected signal only; not FDR-significant. |
| Rook adjacency | Yakeshi City | None | 0 | 0 | -0.60 | 0.724 | 0.056 | 0.917 | No annual upper-tail Gi* signal at uncorrected p < 0.05. |
| Rook adjacency | Morin Dawa Daur Autonomous Banner | None | 0 | 0 | 0.33 | 0.372 | -0.111 | 0.754 | No annual upper-tail Gi* signal at uncorrected p < 0.05. |
| Rook adjacency | Oroqen Autonomous Banner | None | 0 | 0 | -0.97 | 0.833 | -0.111 | 0.754 | No annual upper-tail Gi* signal at uncorrected p < 0.05. |
| Rook adjacency | Ewenki Autonomous Banner | None | 0 | 0 | 0.81 | 0.209 | -0.111 | 0.754 | No annual upper-tail Gi* signal at uncorrected p < 0.05. |
| Rook adjacency | Arun Banner | None | 0 | 0 | 0.10 | 0.460 | -0.167 | 0.602 | No annual upper-tail Gi* signal at uncorrected p < 0.05. |
| Rook adjacency | Erguna City | None | 0 | 0 | -0.33 | 0.629 | 0.278 | 0.348 | No annual upper-tail Gi* signal at uncorrected p < 0.05. |
| KNN=3 | Erguna City | None | 0 | 0 | -0.14 | 0.556 | 0.222 | 0.466 | No annual upper-tail Gi* signal at uncorrected p < 0.05. |

Note: Rook, inverse-distance, and KNN=3 matrices were evaluated on the 2016–2024 county-year incidence panel.

Note: No county-year unit remained significant after FDR correction under any tested spatial-weight matrix.

Note: Uncorrected signals are exploratory and should not be interpreted as confirmatory hotspots.

Supplementary Table S13. Forecasting horizon-level error diagnostics in rolling-origin validation.

| Model | Horizon month | Number of evaluated folds | MAE | RMSE | MAPE (%) | Mean error | Notes |
| --- | --- | --- | --- | --- | --- | --- | --- |
| SARIMA | 1 | 4 | 25.86 | 27.70 | 30.85 | 8.08 |  |
| SARIMA | 2 | 4 | 12.03 | 15.09 | 12.73 | -3.26 |  |
| SARIMA | 3 | 4 | 4.81 | 5.74 | 3.57 | -4.01 |  |
| SARIMA | 4 | 4 | 31.15 | 35.90 | 19.06 | 4.94 |  |
| SARIMA | 5 | 4 | 37.24 | 39.35 | 27.08 | -11.61 |  |
| SARIMA | 6 | 4 | 45.91 | 50.31 | 30.50 | -12.29 |  |
| SARIMA | 7 | 4 | 33.12 | 34.90 | 28.86 | -8.10 |  |
| SARIMA | 8 | 4 | 24.24 | 28.74 | 25.69 | 6.77 |  |
| SARIMA | 9 | 4 | 13.00 | 18.03 | 17.09 | 3.44 |  |
| SARIMA | 10 | 4 | 14.68 | 17.28 | 23.11 | 0.44 |  |
| SARIMA | 11 | 4 | 11.42 | 14.05 | 17.23 | -2.59 |  |
| SARIMA | 12 | 4 | 23.99 | 24.62 | 40.82 | -2.48 |  |
| Prophet | 1 | 4 | 35.89 | 45.76 | 51.60 | -24.76 |  |
| Prophet | 2 | 4 | 30.63 | 34.10 | 31.70 | -28.01 |  |
| Prophet | 3 | 4 | 28.12 | 32.22 | 21.43 | -28.12 |  |
| Prophet | 4 | 4 | 26.38 | 27.55 | 16.56 | -6.33 |  |
| Prophet | 5 | 4 | 35.07 | 37.78 | 25.85 | -35.07 |  |
| Prophet | 6 | 4 | 30.71 | 37.54 | 22.11 | -20.66 |  |
| Prophet | 7 | 4 | 30.95 | 35.44 | 28.74 | -28.83 |  |
| Prophet | 8 | 4 | 22.59 | 30.02 | 27.44 | -22.59 |  |
| Prophet | 9 | 4 | 25.62 | 32.85 | 42.97 | -25.62 |  |
| Prophet | 10 | 4 | 36.45 | 43.09 | 72.76 | -36.45 |  |
| Prophet | 11 | 4 | 36.55 | 40.63 | 50.60 | -36.55 |  |
| Prophet | 12 | 4 | 40.55 | 48.65 | 77.69 | -38.25 |  |
| LightGBM | 1 | 4 | 31.56 | 32.22 | 35.12 | 15.25 | All lagged target-year features were recursively updated from previous predictions. |
| LightGBM | 2 | 4 | 13.63 | 16.56 | 12.55 | -13.34 | All lagged target-year features were recursively updated from previous predictions. |
| LightGBM | 3 | 4 | 20.13 | 21.41 | 15.05 | -13.51 | All lagged target-year features were recursively updated from previous predictions. |
| LightGBM | 4 | 4 | 26.13 | 32.79 | 15.81 | 5.77 | All lagged target-year features were recursively updated from previous predictions. |
| LightGBM | 5 | 4 | 24.65 | 25.60 | 17.70 | -5.19 | All lagged target-year features were recursively updated from previous predictions. |
| LightGBM | 6 | 4 | 19.80 | 27.00 | 14.49 | -13.76 | All lagged target-year features were recursively updated from previous predictions. |
| LightGBM | 7 | 4 | 46.15 | 52.30 | 41.38 | -36.21 | All lagged target-year features were recursively updated from previous predictions. |
| LightGBM | 8 | 4 | 45.95 | 55.78 | 53.87 | -27.84 | All lagged target-year features were recursively updated from previous predictions. |
| LightGBM | 9 | 4 | 30.09 | 38.52 | 48.38 | -20.56 | All lagged target-year features were recursively updated from previous predictions. |
| LightGBM | 10 | 4 | 16.95 | 19.72 | 32.35 | -16.95 | All lagged target-year features were recursively updated from previous predictions. |
| LightGBM | 11 | 4 | 8.06 | 11.52 | 12.27 | -1.90 | All lagged target-year features were recursively updated from previous predictions. |
| LightGBM | 12 | 4 | 16.47 | 18.30 | 31.39 | -7.46 | All lagged target-year features were recursively updated from previous predictions. |

Note: For LightGBM, all lagged features within the forecast year were updated recursively using previous predictions rather than observed target-year values.

Note: The 2025 fold was retained only as a provisional benchmark because of the high deletion rate in 2025 records.

Note: Horizon-level errors are diagnostic and should not be interpreted as deterministic month-specific forecasting accuracy.

Supplementary Table S14. Prediction interval coverage in rolling-origin validation.

| Model | Validation year | Provisional fold | Months evaluated | Months within 95% PI | Coverage (%) | Mean PI width | Median PI width | Notes |
| --- | --- | --- | --- | --- | --- | --- | --- | --- |
| SARIMA | 2022 | No | 12 | 12 | 100.00 | 155.47 | 160.96 | SARIMA(1, 0, 1)(1, 1, 1, 12); lower bound truncated at 0 |
| SARIMA | 2023 | No | 12 | 12 | 100.00 | 143.32 | 144.29 | SARIMA(1, 0, 1)(1, 1, 1, 12); lower bound truncated at 0 |
| SARIMA | 2024 | No | 12 | 12 | 100.00 | 143.28 | 149.28 | SARIMA(1, 0, 1)(1, 1, 1, 12); lower bound truncated at 0 |
| SARIMA | 2025 | Yes | 12 | 12 | 100.00 | 141.34 | 146.97 | SARIMA(1, 0, 1)(1, 1, 1, 12); lower bound truncated at 0 |
| SARIMA | Overall | Includes 2025 provisional fold | 48 | 48 | 100.00 | 145.85 | 149.75 | Limited interval diagnostic across rolling-origin folds. |

Note: The 2025 fold was marked provisional because of the high deletion rate in 2025 records.

Note: Interval outputs were not available or not comparable for all models; Prophet and LightGBM were retained as point-forecast comparisons.

Note: Coverage results are limited interval diagnostics and do not by themselves demonstrate full calibration.

Supplementary Table S15. Software versions and key reproducibility settings.

| Component | Software/package | Version | Key settings | Random seed |
| --- | --- | --- | --- | --- |
| Data processing and tabulation | Python; pandas; numpy | Python 3.13.9; pandas 2.3.3; numpy 2.3.5 | Data cleaning, date conversion, county-year aggregation, incidence calculation, and table generation | Not applicable |
| Spatial data handling | geopandas; GADM boundary data | Version not recorded; GADM v4.1 boundary data | County boundary linkage, adjacency construction, and centroid-based distance calculation | Not applicable |
| Local Gi* and trend diagnostics | Custom Python workflow | Final analysis script | One-sided upper-tail Gi* statistics; Benjamini-Hochberg FDR correction across 117 county-year tests; Mann-Kendall trend test | Not applicable |
| Spatiotemporal scan statistic | Custom Python workflow | Final analysis script | Population-proportional expected counts; single-year and consecutive 2-year windows; 9,999 Monte Carlo simulations; annual total cases fixed | 20260611 |
| SARIMA forecasting | statsmodels | 0.14.5 | Rolling-origin validation; SARIMA seasonal monthly specification; 95% prediction intervals with lower bounds truncated at zero | Not applicable |
| Prophet forecasting | prophet | Version not recorded | Rolling-origin 12-month-ahead point forecasts | Not applicable |
| LightGBM forecasting | lightgbm | Version not recorded | Lag 1–12 features; recursive multi-step forecasting without target-year observed values | 42 |
| Bayesian BYM modeling | PyMC | 5.25.1 | 4 chains; 2,000 warm-up and 2,000 post-warm-up draws per chain; target_accept = 0.99; maximum tree depth = 15; rho sensitivity analyses | 20260507 |
| Bayesian diagnostics | ArviZ | 0.23.4 | Rhat, bulk ESS, tail ESS, divergent transitions, and maximum-tree-depth diagnostics | Not applicable |
| DALY scenario analysis | Python; numpy; pandas | Same as above | YLD = incident cases × disability weight × duration; YLL set to zero because mortality linkage was unavailable | Not applicable |

Note: This table summarizes the main software packages and model settings used for data processing, spatial analysis, forecasting, Bayesian modeling, and sensitivity analyses. Package versions are reported where available from the final analysis environment. Random seeds are provided for stochastic procedures where applicable.

Supplementary Table S16. Forecasting sensitivity analysis excluding the provisional 2025 fold.

| Analysis set | Validation years | Model | Folds/months | MAE | RMSE | MAPE (%) | Mean annual total error (%) | Interpretation |
| --- | --- | --- | --- | --- | --- | --- | --- | --- |
| All rolling-origin folds | 2022–2025 | SARIMA | 4/48 | 23.12 | 28.68 | 23.05 | 14.01 | Lowest monthly MAE and MAPE across all folds. |
| All rolling-origin folds | 2022–2025 | Prophet | 4/48 | 31.62 | 37.64 | 39.12 | 25.19 | Higher errors in this local monthly series. |
| All rolling-origin folds | 2022–2025 | LightGBM | 4/48 | 24.96 | 32.17 | 27.53 | 13.74 | Marginally lowest mean annual total error; recursive multi-step forecasts. |
| Excluding provisional 2025 fold | 2022–2024 | SARIMA | 3/36 | 23.52 | 29.05 | 22.46 | 12.45 | Lowest monthly MAE, MAPE, and mean annual total error after excluding 2025. |
| Excluding provisional 2025 fold | 2022–2024 | Prophet | 3/36 | 34.44 | 40.34 | 42.91 | 27.29 | Higher errors in this local monthly series. |
| Excluding provisional 2025 fold | 2022–2024 | LightGBM | 3/36 | 26.16 | 34.34 | 29.07 | 13.11 | Annual total error remained close to SARIMA, but monthly errors were higher. |

Note: Metrics were recalculated from the rolling-origin validation detail file. The sensitivity set excludes the 2016–2024 training / 2025 validation fold because the 2025 surveillance file had a high deletion rate. LightGBM forecasts used recursive lag updating, so no target-year observed values were used as lagged predictors within the forecast horizon.
